# Multi‐Agent‐Network‐Based Idea Generator for Zinc‐Ion Battery Electrolyte Discovery: A Case Study on Zinc Tetrafluoroborate Hydrate‐Based Deep Eutectic Electrolytes

**DOI:** 10.1002/adma.202502649

**Published:** 2025-05-22

**Authors:** Matthew J. Robson, Shengjun Xu, Zilong Wang, Qing Chen, Francesco Ciucci

**Affiliations:** ^1^ Department of Mechanical and Aerospace Engineering The Hong Kong University of Science and Technology Kowloon Hong Kong SAR China; ^2^ University of Bayreuth Chair of Electrode Design for Electrochemical Energy Systems 95448 Bayreuth Germany; ^3^ University of Bayreuth Bavarian Center for Battery Technology (BayBatt) Universitätsstraße 30 95447 Bayreuth Germany; ^4^ The Energy Institute The Hong Kong University of Science and Technology Clear Water Bay Kowloon Hong Kong SAR China; ^5^ Department of Chemistry The Hong Kong University of Science and Technology Clear Water Bay Kowloon Hong Kong SAR China

**Keywords:** batteries, eutectic electrolytes, large language models

## Abstract

Aqueous deep eutectic electrolytes (DEEs) offer great potential for low‐cost zinc‐ion batteries but often have limited performance. Discovering new electrolytes is therefore crucial, yet time‐consuming and resource‐intensive. In response, this work presents a Large Language Model (LLM)‐based multi‐agent network that proposes DEE compositions for zinc‐ion batteries. By analyzing academic papers from the DEE field, the network identifies innovative, inexpensive, and sustainable Lewis bases to pair with Zn(BF_4_)_2_·xH_2_O. A Zn(BF_4_)_2_·xH_2_O‐ethylene carbonate (EC) system demonstrates high conductivity (10.6 mS cm^−1^) and a wide electrochemical stability window (2.37 V). The optimized electrolyte enables stable zinc stripping/plating, achieves outstanding rate performance (81 mAh g^−1^ at 5 A g^−1^), and supports 4000 cycles in Zn||polyaniline cells at 3 A g^−1^. Spectroscopic analyses and simulations reveal that EC coordinates to Zn^2+^_,_ mitigating water‐induced corrosion, while a fluorine‐rich hybrid organic/inorganic solid electrolyte interphase enhances stability. This work showcases a pioneering LLM‐driven approach to electrolyte development, establishing a new paradigm in materials research.

## Graphical abstract

An LLM‐based multi‐agent network screens academic literature to propose multiple environmentally friendly aqueous deep eutectic electrolytes for zinc‐ion batteries. Experiments identify an optimal composition of Zn(BF_4_)_2_·xH_2_O and ethylene carbonate, which shows high conductivity and cycling stability. Spectroscopy and simulations confirm corrosion suppression and stable solid electrolyte interphases, establishing an AI‐driven paradigm for electrolyte discovery.

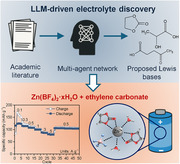

## 1 Introduction

Aqueous zinc‐ion batteries have attracted increasing research attention due to their low cost, environmentally friendly construction, abundant materials, and simple processing.^[^ ^1^ ^]^ However, they are typically limited by the poor electrochemical stability window of water (1.23 V vs H^+^/H) and its tendency to corrode the Zn anode and dissolve common cathode materials. Deep eutectic electrolytes (DEEs) have emerged as a promising solution to these challenges. DEEs comprise a Lewis acid (hydrogen bond donor) and Lewis base (hydrogen bond acceptor) that form strong interspecies hydrogen‐bonding networks when mixed.^[^ ^2^ ^]^ This bonding leads to significant melting point depression and can mitigate the inherent water‐induced corrosion at the zinc anode, expanding the voltage stability window.^[^ ^3^ ^]^ DEEs offer additional advantages such as non‐flammability, low volatility, and wide operating temperature ranges.^[^ ^3^ ^]^ Moreover, eutectic bonding structures demonstrate exceptional tunability, enabling manipulation of zinc‐ion coordination environments to optimize ionic conductivity and stability across various electrolyte systems.^[^ ^4^, ^5^, ^6^ ^]^

Zinc tetrafluoroborate hydrate (Zn(BF_4_)_2_·xH_2_O, ZBF) has emerged as an exciting Lewis acid target for high‐performance, stable zinc batteries with DEEs. The potential of ZBF stems from its low bulk prices and its tendency to form a fluorinated solid electrolyte interphase (SEI) on the Zn anode through the spontaneous decomposition of the BF_4_^−^ anion.^[^ ^7^, ^8^, ^9^ ^]^ This SEI composition is particularly beneficial, as surface fluorination is a widely recognized strategy for enhancing cycling stability in zinc batteries.^[^ ^10^, ^11^, ^12^, ^13^ ^]^ Achieving such an SEI in Zn batteries has typically required the introduction of expensive zinc salts and additives such as zinc bis(trifluorosulfonylimide) (Zn(TFSI)_2_), zinc trifluoromethylsulfonate (Zn(OTf)_2_), and fluoroethylene carbonate.^[^ ^10^, ^14^, ^15^ ^]^ Han et al. first proposed a cost‐effective DEE combining ZBF and ethylene glycol, achieving excellent long‐term cycling performance exceeding 4000 h at 0.5 mA cm^−2^ and 0.25 mAh cm^−2^ in part due to its SEI.^[^ ^7^ ^]^ A subsequent study using ZBF and acetamide revealed a multi‐layered SEI with distinct fluorine‐oxygen and boron‐oxygen‐rich layers. By optimizing the acetamide to ZBF ratio, this electrolyte prevented BF_4_^−^ hydrolysis and zinc corrosion, enabling a Zn cell with a polyaniline (PANI) cathode to achieve 4000 cycles with 73% capacity retention at 1 A g^−1^.^[^ ^8^ ^]^ Despite these promising results, research specifically targeting novel ZBF‐based DEEs remains limited, presenting significant opportunities to enhance performance by exploring alternative Lewis bases.

The remarkable potential of ZBF‐based DEEs is evident, yet the identification of optimal Lewis acid/base combinations presents a significant challenge. This difficulty arises from the large design space of possible compositions, necessitating the exploration of innovative approaches to expedite electrolyte discovery. Conventionally, the selection of electrolyte components has been a time‐consuming and predominantly human‐driven process, relying on creative insights and a deep understanding of the scientific literature, both aspects that are challenging to codify. Recently, the advent of large language models (LLMs) has marked a new era in natural language processing. These models, which are trained on vast unstructured text datasets, have demonstrated excellent performance on open‐domain question answering on a wide variety of topics, such as language translation, text summarization, medical diagnosis assistance, and scientific literature analysis, along with a promising capacity to assist in generating scientific hypotheses.^[^ ^16^, ^17^, ^18^, ^19^, ^20^ ^]^ However, LLMs can produce inaccurate responses that can be observed when queried on specific topics due to flawed data sources, training inconsistencies, and defective decoding strategies.^[^ ^21^, ^22^, ^23^ ^]^ Retrieval augmented generation (RAG) techniques offer a promising avenue for enhancing response accuracy. These methods often employ a “retrieve‐then‐read” pipeline, utilizing similarity search algorithms to analyze provided text documents and extract relevant contextual information.^[^ ^24^ ^]^ Leveraging RAG to enhance LLM performance, researchers have successfully generated novel targets for drug discovery and accurately predicted outcomes of metal–organic framework synthesis experiments.^[^ ^18^, ^19^ ^]^ However, this approach has never been applied to the development of novel battery electrolytes.

Beyond single‐ and multi‐shot approaches to open‐domain question answering, LLMs have shown enhanced creativity and accuracy when augmented with agent‐based frameworks.^[^ ^25^, ^26^, ^27^ ^]^ These agents, capable of hypothesis generation, external information access, and iterative course correction, have been successfully deployed as chemistry assistants and experiment planners.^[^ ^28^, ^29^ ^]^ Such enhancements likely arise from increased specialization, task decomposition, and the diverse perspectives afforded by varying prompts and LLMs across agents.^[^ ^27^ ^]^ Conversational multi‐agent networks (MANs) further expand the capabilities of chat‐focused LLMs in complex tasks.^[^ ^30^, ^31^ ^]^ Each agent in the network leverages RAG to access and process relevant information from their designated knowledge bases, while the multi‐agent architecture enables iterative feedback from other agents and refinement of these retrievals. This approach can mitigate the error propagation often associated with LLM chaining.^[^ ^27^ ^]^ Notably, Wu et al.’s AutoGen framework provided a flexible architecture for defining agent interactions, surpassing both ReAct agents and ChatGPT with plugins in mathematical problem‐solving and RAG tasks.^[^ ^31^ ^]^ Building on this nascent potential, we present the first application of a multi‐agent approach for novel electrolyte composition discovery, focusing on ZBF‐based DEEs for zinc‐ion batteries. In this study, we leverage a novel MAN approach utilizing LLMs and RAG techniques to expedite the discovery of ZBF‐based DEEs for zinc‐ion batteries, aiming to overcome the challenges of conventional electrolyte development and unlock new frontiers in high‐performance, cost‐effective energy storage solutions.

## 2 Results and Discussion

**Figure 1:**
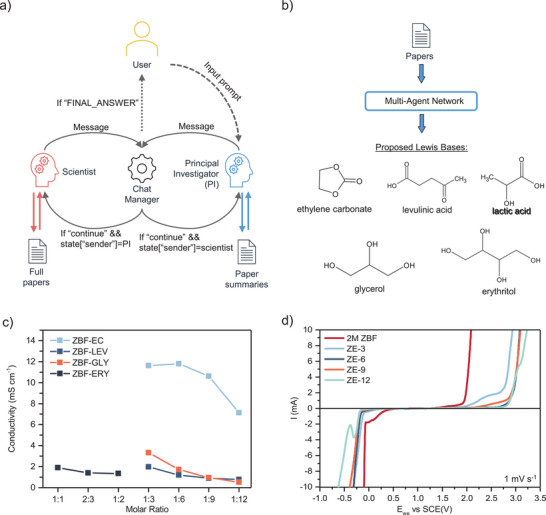
a) Schematic of the multi‐agent network used for electrolyte discovery and b) the proposed Lewis bases. c) Room‐temperature conductivity screening of electrolytes based on ZBF paired with ethylene carbonate (ZBF‐EC), glycerol (ZBF‐GLY), levulinic acid (ZBF‐LEV), and erythritol (ZBF‐ERY). Electrolytes were only tested at molar ratios where they remained fully liquid. d) Electrochemical windows of ZBF‐EC electrolytes at various molar ratios (denoted ZE‐x, where x represents the molar ratio of EC to ZBF), compared to a 2 m ZBF reference.

The MAN schematized in **Figure** **1**a was used as an idea generator to propose novel electrolyte components for ZBF‐based deep eutectic electrolytes. To achieve this, a database of articles on DEEs for Zn batteries (Table S1, Supporting Information) was compiled and processed using the GPT‐4 model to produce focused summaries, one per paper. The input database of papers, prompts, and detailed processing information can be found in the Supporting Information and Prompts S1–S5 (Supporting Information). A multi‐agent network constructed using LangChain and LangGraph employed two AI agents: a “Principal Investigator” accessing concise paper summaries to identify broad trends and potential novel connections across the literature, and a “Scientist” accessing detailed full papers for validation and concept refinement.^[^ ^32^, ^33^ ^]^ This dual‐agent architecture was selected based on principles observed in effective human research teams and multi‐agent AI systems, aiming to enhance the robustness and creativity of the ideation process.^[^ ^27^, ^34^, ^35^ ^]^ These agents, utilizing separate instances of the GPT‐4 model, employed specific information retrieved from the provided literature to ground and direct the generative capabilities offered by the model's vast pre‐trained knowledge base, leading to the proposal of new DEE compositions. First, an exploratory prompt designed to identify novel chemical combinations with high compositional freedom was used (Prompt S4, Supporting Information), and second, a focused prompt was employed to investigate novel ZBF‐based systems (Prompt S5, Supporting Information) systematically. The targeted approach with ZBF was built upon the documented success of this compound in prior DEE applications.^[^ ^7^, ^8^ ^]^

Using these prompts, the MAN rapidly generated innovative potential DEE compositions (Tabulated in Table S2, Supporting Information), with an average generation time of 234 s per candidate. An example conversation between the agents is provided in the Supporting Information, which demonstrates how the RAG component actively steered the ideation, grounding the LLM's generative capabilities in domain‐specific findings from the literature. This MAN approach excels in efficiency and accessibility, requiring minimal setup time and expert involvement as it needs only relevant academic papers as input, a process potentially automatable through web scraping. The average time taken to extract and prepare summaries of each paper was 42 s. A further 40 s were required to create the full‐text vector database and 4 s to create the summary vector database for all 79 papers. While direct quantitative comparison with the highly variable human research process is inherently challenging, this computational ideation time represents a significant acceleration compared to the weeks or months potentially required for a comparable human‐led literature review and hypothesis generation. Importantly, the agent and input prompts can be tailored to specific applications, making the approach highly versatile. The MAN's ability to rapidly process information from academic literature and propose candidates within minutes, combined with its natural language processing capabilities and broad knowledge base, may, therefore, enable it to rapidly identify subtle connections and generate novel ideas that might be overlooked by conventional expert‐guided database searches or simulations. However, it is essential to clarify that the MAN leverages RAG primarily for conceptual synthesis and identifying qualitative trends from the text, rather than attempting to quantitatively validate or reconcile potentially inconsistent numerical data reported across different studies. In addition, while the system can propose candidate compositions, current LLM limitations prevent accurate prediction of optimal concentration ranges or eutectic points. These critical parameters still require experimental determination due to the complex, composition‐specific nature of phase behavior and solvation thermodynamics.

To systematically evaluate the proposed compositions, only novel Lewis base pairings with ZBF were selected for experimental analysis. This means that Lewis acids such as choline acetate and LiTFSI were excluded, along with H_2_O, which is already present in ZBF. This approach uncovered several promising candidates, including cost‐effective (Figure S2, Supporting Information) and environmentally friendly biologically derived compounds. Notably, erythritol and levulinic acid emerged as intriguing options. While these compounds have been previously demonstrated as viable Lewis bases for deep eutectic solvents in applications such as CO_2_ capture and chemical extraction,^[^ ^36^, ^37^, ^38^ ^]^ they have not been explored in Zn battery applications. This innovative repurposing of materials from other fields underscores the MAN's creativity. Similarly, the potential of ethylene carbonate (EC) as a Lewis base in DEEs for zinc batteries remains unexplored despite its proven ability to regulate deposition in aqueous zinc batteries through SEI formation.^[^ ^39^, ^40^ ^]^ The electrolyte systems selected for testing were, therefore, ZBF and erythritol (ZBF‐ERY), ZBF and glycerol (ZBF‐GLY), ZBF and lactic acid (ZBF‐LAC), ZBF and ethylene carbonate (ZBF‐EC), and ZBF and levulinic acid (ZBF‐LEV). All systems formed liquid‐phase electrolytes at different molar ratios, which are tabulated in Table S3 (Supporting Information).

The proposed electrolyte systems were then screened using electrochemical impedance spectroscopy (EIS) to determine electrolyte conductivity and linear sweep voltammetry to ascertain the electrochemical window (Figure 1c; Figures S4 and S5, Supporting Information). The ZBF‐LAC system was found to aggressively corrode both the Zn anode and stainless steel (SS), as could be expected for an acidic electrolyte, and thus gave unstable EIS data (Figure S4e, Supporting Information). Of the tested systems, ZBF‐EC demonstrated the highest conductivity of 11.8 mS cm^−1^ at a ratio of 1:6, decreasing to 10.6 mS cm^−1^ at a 1:9 ratio (Figure 1c). This conductivity exceeds the performance of existing ZBF‐based DEE systems by a factor of 2 (Table S4, Supporting Information). The ZBF‐EC system was also able to achieve a wide electrochemical stability window of ≈2.4 V at a molar ratio of 1:9 ZBF:EC (Figure 1d), greatly expanding the voltage window against an aqueous 2 m ZBF electrolyte. Considering its superior performance, the ZBF‐EC electrolyte system was selected for further analysis. Electrolytes, denoted ZE‐x (x = 3, 6, 9, 12), representing ZBF:EC molar ratios of 1:*x*, were prepared. DEE formation was quantitatively confirmed by differential scanning calorimetry (DSC) on ZE‐9, which showed substantial freezing point suppression in comparison to pure EC (Figure S6, Supporting Information). Electrolyte ionic conductivity with temperature was also tested for the ZE‐x system, demonstrating substantial improvements in low‐temperature performance with higher proportions of ZBF (Figure S7, Supporting Information). Flammability tests were also performed on fiberglass separators soaked in ZE‐9 and pure EC (Figure S8, Supporting Information). When exposed to a naked flame in air, ZE‐9 demonstrated self‐extinguishing behavior. In contrast, a separator soaked in EC sustained a flame once ignited. The self‐extinguishing nature of ZE‐9 is attributed to the electrolyte's water content and the characteristically low vapor pressures of DEEs.

**Figure 2:**
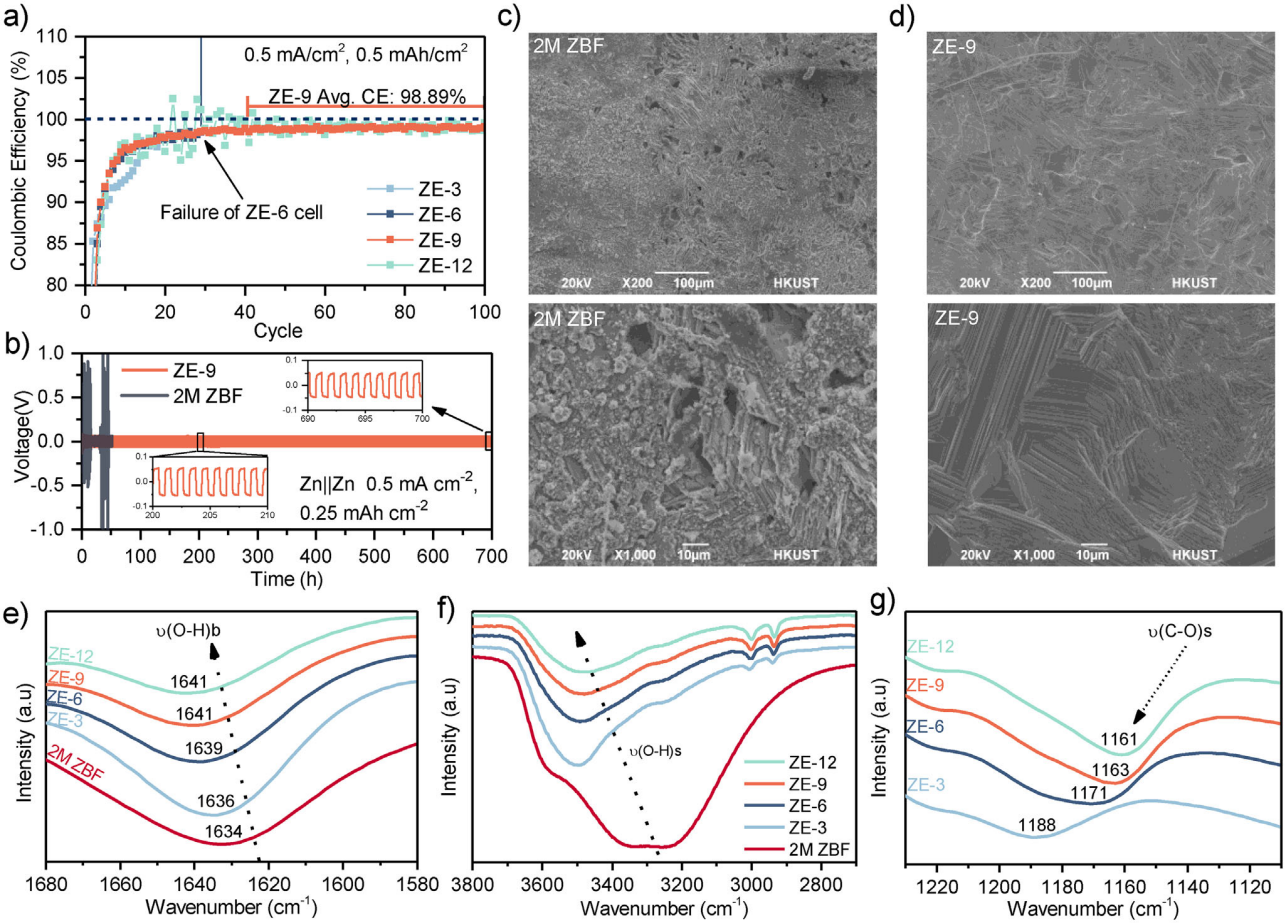
a) Plot of Coulombic efficiency against cycle number from Zn|ZE‐x|SS cells. b) Voltage profiles from a Zn||Zn symmetric cell cycling at 0.5 mA cm^−2^ and 0.25 mAh cm^−2^. SEM images of as‐cycled Zn anodes from Zn||Zn symmetric cells after 50 cycles at 0.5 mA cm^−2^ and 0.25 mAh cm^−2^ with c) 2 m ZBF and d) ZE‐9 electrolytes. FTIR plots of the e) O─H bending, f) O─H stretching, and g) C─O stretching vibration peaks.

The Coulombic efficiency of ZE‐x electrolytes was evaluated through cycling tests in Zn||SS cells at 0.5 mA cm^−2^ and 0.5 mAh cm^−2^ (**Figure** **2**a). While ZE‐3, ZE‐6, and ZE‐12 electrolytes exhibited unstable Coulombic efficiencies during plating and stripping, ZE‐9 demonstrated remarkable stability. Notably, ZE‐6, despite its higher conductivity (11.8 mS cm⁻¹) and similar electrochemical stability window compared to ZE‐9 (Figure 1c,d), showed poor cycling performance, failing after only 29 cycles. In contrast, ZE‐9 cycled stably 100 times, achieving an average Coulombic efficiency of 98.89%, which surpasses previously reported values for aqueous DEEs in Zn||SS cells (Table S5, Supporting Information). This high Coulombic efficiency is particularly notable given that SS substrates are more susceptible to competitive hydrogen evolution and corrosion reactions in aqueous systems, typically reducing the Coulombic efficiency compared to more stable Zn||Cu cells.^[^ ^41^ ^]^ The ZE‐9 electrolyte also sustained cycling for over 700 h in a Zn||Zn symmetric cell at 0.5 mA cm^−2^ and 0.25 mAh cm^−2^, whereas the Zn|2 m ZBF|Zn cell exhibited unstable plating and stripping, ultimately short‐circuiting after 52 h. The exceptional performance of Zn|ZE‐9|Zn cells was further evidenced by their ability to withstand over 1000 cycles at 0.2 mA cm^−2^ and 0.1 mAh cm^−2^, underscoring the electrolyte's compatibility with the Zn anode (Figure S9, Supporting Information). Considering its superior conductivity, Coulombic efficiency, and stable symmetric cell cycling performance, ZE‐9 emerged as the optimal electrolyte within the ZE‐x series.

Scanning electron micrographs (SEM) of as‐cycled anodes from cells cycled with the comparison 2 m ZBF electrolyte (Figure 2c) showed a rough, inhomogeneous deposition morphology, with mossy Zn deposits and corrosion pores visible across the surface. In addition, large, single crystals embedded into the anode surface were observed, which were identified to be highly fluorine‐rich by energy‐dispersive X‐ray spectroscopy (EDX) analysis (Figure S10, Supporting Information). In contrast, Zn anodes cycled in the ZE‐9 electrolyte (Figure 2d) had a smooth and regular morphology devoid of mossy deposits. The surface presented aligned striations indicative of ordered Zn deposition, suggesting stable stripping and plating behavior.^[^ ^42^ ^]^ EDX analysis of the cycled Zn anode revealed the homogeneous presence of carbon and fluorine on the surface, implying the formation of an SEI produced by the decomposition of EC and BF_4_^−^ (Figure S11, Supporting Information).

To further assess DEE compatibility with the anode, Zn foil was soaked in 2 m ZBF and ZE‐9 electrolytes for 7 days, with optical images presented in Figure S12 (Supporting Information). The Zn sample immersed in 2 m ZBF exhibited a rough surface covered with thick, heterogeneous crystalline deposits, which X‐ray diffraction (XRD) analysis identified as predominantly zinc hydroxyfluoride (Zn(OH)F) (Figure S13, Supporting Information). These crystals likely formed due to the spontaneous hydrolysis of tetrafluoroborate (BF_4_^−^) in aqueous media, as previously reported.^[^ ^43^ ^]^ In contrast, the ZE‐9 electrode, while discolored compared to the bare Zn reference, showed no visible crystalline deposits. Moreover, the XRD spectrum revealed no additional reflections beyond those of pure Zn (Figure S14, Supporting Information). These findings suggest that the addition of EC inhibited the hydrolysis of BF_4_^−^, preventing electrode corrosion.

To elucidate the mechanism underlying the stable cycling behavior, we conducted Fourier transform infrared (FTIR) spectroscopic analysis on the ZE‐x and 2 m ZBF electrolytes and their components (Figure 2e–g; Figure S15, Supporting Information). FTIR analysis revealed a progressive red shift in the O─H bending vibration peak from 1641 to 1634 cm^−1^ as EC concentration decreased from ZE‐9/ZE‐12 to 2 m ZBF, indicating stronger water bonding in electrolytes with higher EC content (Figure 2e).^[^ ^44^, ^45^ ^]^ The O─H stretching regime exhibited significant narrowing compared to the 2 m ZBF reference electrolyte (Figure 2f), suggesting a reduction in the diversity of H_2_O bonding environments and a concomitant decrease in free water clusters.^[^ ^44^ ^]^ Additionally, a pronounced red shift in the C─O stretching vibration band was observed with increasing Zn^2+^ concentration (Figure 2g), indicative of robust EC complexation around Zn^2+^ ions.^[^ ^46^ ^]^ Collectively, these spectroscopic findings suggest that EC preferentially coordinates with Zn^2+^, forming a strong primary solvation shell with a reduced number of water molecules directly coordinated with Zn^2+^. This modified solvation environment suppresses corrosion by limiting water availability for zinc hydroxide formation at the metal surface.^[^ ^45^, ^47^ ^]^

**Figure 3:**
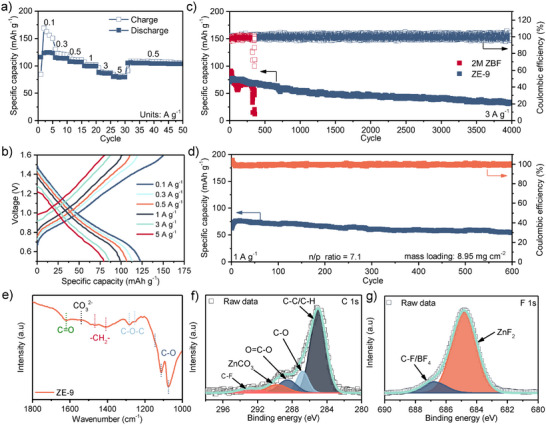
a) Rate performance and b) associated specific capacity against voltage plots for the Zn|ZE‐9|PANI cells. c) Long‐term cycling performance of Zn|ZE‐9|PANI cells and Zn|2 m ZBF|PANI cells at 3 A g^−1^. d) Cycling performance of Zn|ZE‐9|PANI cells at 1 A g^−1^ with a low n/p ratio of 7.1. e) FTIR spectrum, and XPS spectra from the f) C 1s and g) F 1s orbitals collected from the surface of Zn anodes cycled in Zn|ZE‐9|PANI cells.

The full‐cell performance of the ZE‐9 electrolyte was evaluated using PANI cathodes. Zn|ZE‐9|PANI cells exhibited exceptional rate performance, delivering specific capacities of 124, 114, 107, 102, 88, and 81 mAh g^−1^ at current densities of 0.1, 0.3, 0.5, 1, 3, and 5 A g^−1^, respectively (**Figure** **3**a,b). This rate capability surpasses that of other binary hydrated deep eutectic electrolytes reported in the literature (Table S4, Supporting Information). Upon returning to 0.5 A g^−1^, the specific capacity recovered to 105 mAh g^−1^, demonstrating excellent reversibility. Long‐term cycling tests of Zn|ZE‐9|PANI cells revealed high initial capacities of 78 mAh g^−1^ and remarkable cycling stability, retaining ≈57% capacity after 2500 cycles and ≈43% after 4000 cycles at 3 A g^−1^ (Figure 3c). By comparison, while the Zn|2 m ZBF|PANI cells showed higher initial capacities of 92 mAh g^−1^, they exhibited unstable cycling behavior, experiencing a concurrent, sharp decrease in Coulombic efficiency and specific capacity after only 354 cycles at 3 A g^−1^. The enhanced initial performance of the Zn|2 m ZBF|PANI cell can be attributed to the greater presence of water, which not only provides higher conductivity but may also enhance ion transport and active material utilization within the PANI cathode. However, the water content also promotes BF_4_⁻ hydrolysis, which results in HF formation, along with additional side reactions that lead to rapid capacity fading.^[^ ^43^ ^]^ To further demonstrate the robustness of the ZE‐9 system, we tested a Zn|ZE‐9|PANI cell with a higher cathode mass loading of 8.95 mg cm^−2^ and a negative/positive mass ratio of 7.1. This cell maintained ≈72% capacity retention over 600 cycles with a 99.86% Coulombic efficiency, underscoring the excellent stability of the ZE‐9 electrolyte under more demanding conditions.

To determine the composition of the solid‐electrolyte interphase formed during cycling, anode surfaces from Zn|ZE‐9|PANI cells cycled at 3 A g^−1^ for 100 cycles were characterized with FTIR and X‐ray photoelectron spectroscopy (XPS). FTIR spectroscopy results (Figure 3e) reveal a complex organic layer on the Zn anode surface, characterized by distinct peaks at 1074, 1111, and 1146 cm^−1^. These peaks are attributable to C─O stretching vibrations in alkyl carbonate salts, alkyl carbonates, and polyethylene oxide (PEO)‐type polymers, respectively.^[^ ^40^, ^48^ ^]^ Additional peaks at 1409 and 1466 cm^−1^ were attributed to CH_3_/CH_2_ group stretching, while those at 1280 and 1253 cm^−1^ corresponded to C─O─C bond vibrations.^[^ ^40^, ^48^ ^]^ A low‐intensity, broad peak at ≈1539 cm^−1^ was also present, indicative of CO_3_^2−^ species and consistent with other alkyl carbonate electrolytes.^[^ ^48^, ^49^ ^]^

Analysis of the F 1s XPS spectrum (Figure 3g) reveals a primary peak at 685.0 eV and a secondary shoulder at 686.8 eV, attributable to ZnF_2_ and F in C‐F or BF_4_^−^ species, respectively.^[^ ^7^, ^50^ ^]^ Examination of the C 1s spectrum (Figure 3f) indicates carbon in multiple bonding environments, with peaks at 285.0, 286.8, and 288.6 eV corresponding to C─C/C─H, C─O, and O═C─O bonds, respectively.^[^ ^40^, ^50^ ^]^ Additionally, a subpeak at 289.8 eV is associated with ZnCO_3_,^[^ ^40^ ^]^ while a weak signal at 292.9 eV suggests the presence of C─F bonds.^[^ ^50^ ^]^ The Zn 2p_3/2_ and B 1s spectra are presented in Figure S16a,b (Supporting Information), respectively. Zn 2p_3/2_ signal can be deconvoluted into 3 peaks corresponding to Zn^0^ and Zn^i^ and Zn^ii^, which represent zinc metal, zinc oxide, and higher order zinc compounds, such as ZnF_2_, Zn(OH)F, and Zn(OH)_2_.^[^ ^7^ ^,44,51]^ The location of the primary peak in the B 1s spectrum is consistent with B─O and B─F bonding.^[^ ^8^, ^52^ ^]^ These findings suggest the concurrent adsorption and decomposition of the BF_4_^−^ anion and EC on the Zn surface, resulting in the formation of a ZnF_2_‐rich SEI, along with the incorporation of various alkyl carbonate and fluorinated polymers. This composition indicates the formation of an inorganic/organic hybrid solid electrolyte interphase, consistent with previous reports on aqueous Zn batteries containing EC and other ZBF‐based DEEs.^[^ ^7^, ^8^, ^40^ ^]^ The apparent synergistic effect of these hybrid inorganic/organic decomposition products likely passivates the Zn surface, suppressing corrosion and regulating the Zn deposition morphology. In agreement with previous ZBF‐ and EC‐containing electrolytes in Zn batteries, this passivation film inhibits dendrite nucleation and growth, thereby enhancing the cycling stability of the battery.^[^ ^7^, ^40^ ^]^

**Figure 4:**
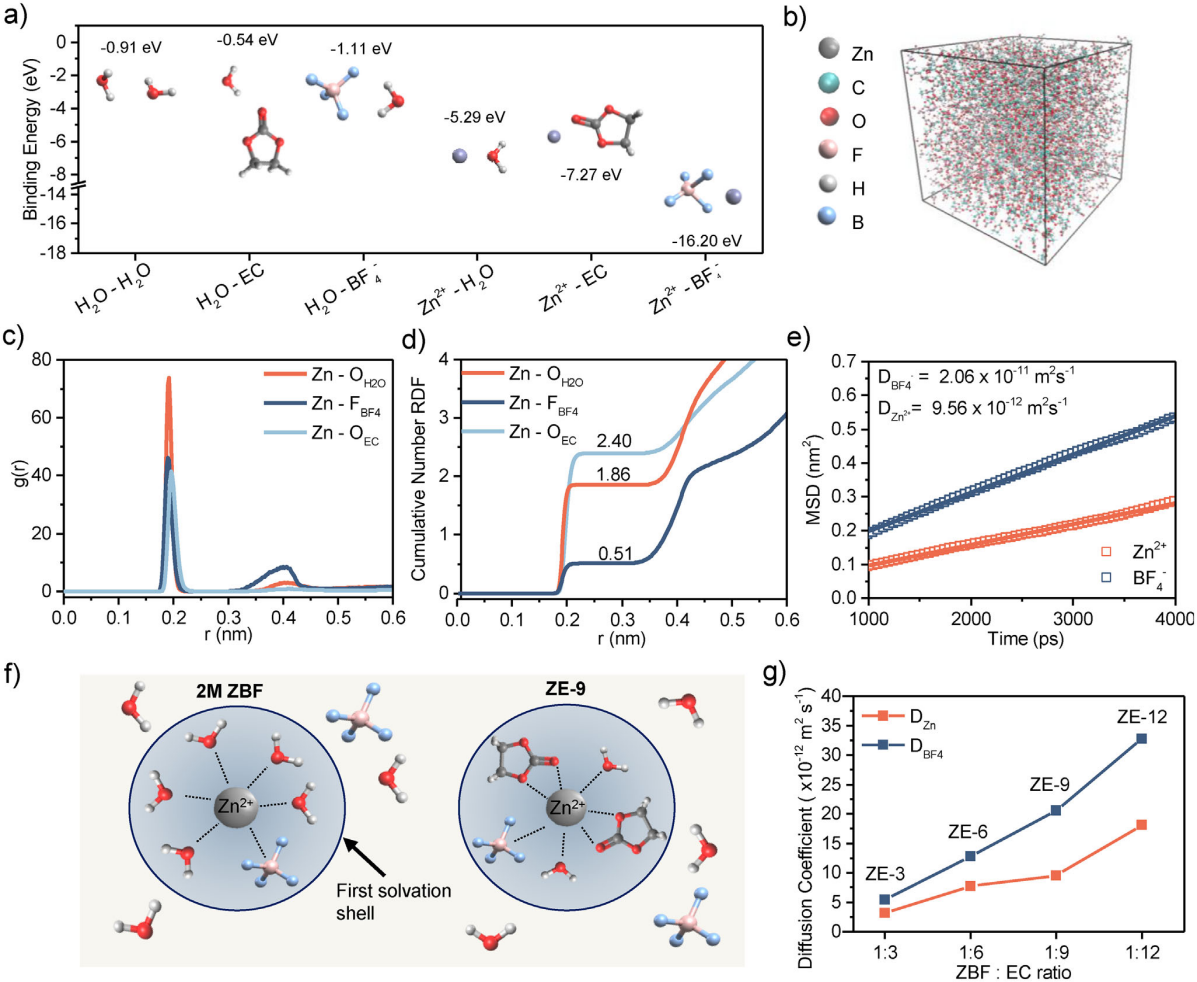
a) Binding energy diagram for Zn^2+^ and EC with other electrolyte components. b) Molecular dynamics snapshot of the ZE‐9 system during production. c) Radial distribution function and d) coordination number of the ZE‐9 electrolyte system. e) Mean squared displacement of Zn^2+^ and BF_4_^−^ in ZE‐9 within the 1000–4000 ps range used to calculate the diffusion coefficient. f) Schematic diagram showing solvation structures of the 2 m ZBF and ZE‐9 electrolytes. g) Comparison of diffusion coefficients in the ZE‐3, ZE‐6, ZE‐9, and ZE‐12 electrolyte systems.

Density functional theory (DFT) calculations and molecular dynamics (MD) simulations were performed to provide insight into the solvation structures formed within the electrolyte. First, DFT was used to assess the binding energies of H_2_O and Zn^2+^ with other electrolyte components (**Figure** **4**a). The binding energy of Zn^2+^‐EC was found to be considerably higher than Zn^2+^‐H_2_O, indicating strong, preferential bonding between Zn and EC over water.

MD simulations were then performed on 2 m ZBF, ZE‐3, ZE‐6, ZE‐9, and ZE‐12 model systems, with the simulation snapshots presented in Figure 4b and Figure S17 (Supporting Information). The radial distribution functions around the Zn^2+^ ion reveal first solvation shell peaks at 0.19 nm for BF_4_^−^, and 0.192 nm for EC and H_2_O across all containing systems (Figure 4c; Figure S17, Supporting Information). Coordination analysis of the ZE‐9 system shows average coordination numbers of 2.40, 1.86, and 0.51 for EC, H_2_O, and BF_4_^−^, respectively, around Zn^2+^ (Figure 4d), contrasting with the water‐dominated coordination in the reference 2 m ZBF system (4.95 H_2_O and 0.25 BF_4_^−^).

A clear EC concentration‐dependent evolution of the solvation structure emerges across the ZE‐x series. In ZE‐3, water remains dominant in the first solvation shell (2.47 H_2_O and 1.20 EC per Zn^2+^ ion, Figure S17, Supporting Information), potentially explaining this system's poor cycling stability. As EC content increases in higher ZE‐x systems, EC progressively displaces H_2_O from the Zn^2+^ solvation shell, correlating with enhanced electrolyte stability. This preferential EC coordination prevents the formation of highly corrosive hexaaqua Zn species typically present in aqueous conditions, thereby mitigating water‐induced anodic corrosion.^[^ ^53^ ^]^ The computed solvation structure, illustrated schematically in Figure 4f, is consistent with FTIR observations.

The modified solvation environment also influences ion transport properties. The mean‐squared displacement (MSD) plots of Zn^2+^ and BF_4_^−^ in ZE‐9 are presented in Figure 4e along with the MSD plots for the other ZE‐x systems in Figure S18 (Supporting Information). The diffusion coefficients of Zn^2+^ and BF_4_^−^ are found to increase with increasing proportion of EC in the electrolyte (Figure 4g).

Starting from the average [Zn(EC)_2_(H_2_O)_2_(BF_4_)]^+^ coordination structure inferred from MD analysis for ZE‐9, the desolvation energies for the system were calculated using DFT methods (Figure S19, Supporting Information). The lowest energy desolvation pathway revealed a sequential loss of the two H_2_O molecules to form [Zn(EC)_2_(BF_4_^−^)]^+^. This finding suggests Zn^2+^ and H_2_O interact more weakly in the presence of EC. Notably, the DFT calculations show that EC and BF_4_^−^ maintain stronger bonding with Zn^2+^ throughout most of the simulated desolvation process, which aligns with the experimentally observed inclusion of EC decomposition products and fluorine within the anode SEI.

## 3 Conclusion

These findings underscore the potential of LLMs as innovative idea‐generation tools for battery scientists, specifically in the field of aqueous DEEs for zinc ion batteries. Building upon the promise of ZBF as a Lewis acid in DEEs, a MAN was employed to identify novel Lewis base pairings. Guided by insights from an automated academic literature review and leveraging the LLM's chemical knowledge, this network proposed highly novel electrolyte components such as erythritol, EC, and levulinic acid, which had not been previously explored in this context.

The proposed components were experimentally screened, and the most promising electrolyte system containing ZBF and EC was characterized. The optimal ZE‐9 electrolyte composition demonstrated outstanding conductivity (10.6 mS cm^−1^) and a wide electrochemical stability window (2.39 V). In a Zn|PANI cell, this electrolyte enabled an impressive cycle life of 4000 cycles at a high current density of 3 A g^−1^ and 600 cycles at 1 A g^−1^ with a remarkable 72% capacity retention, even at a low negative/positive mass ratio of 7.1. This excellent performance was attributed to a hybrid organic/inorganic, fluorinated SEI formed by the simultaneous decomposition of EC and BF_4_^−^ on the Zn surface, coupled with the strong coordination between EC and Zn^2+^.

This work strongly illustrates the outstanding potential of multi‐agent LLM tools to augment battery development. Compared to conventional methods, LLM tools significantly accelerate the initial discovery and hypothesis generation phase, thereby reducing the associated time and resource investment. This research highlights a new and rapidly emerging paradigm in battery research, where LLM‐assisted discovery effectively complements traditional experimental characterization, accelerating the development of next‐generation energy storage solutions.

## 4 Experimental Section

### Summary Preparation

A database containing 79 papers from the field of deep eutectic electrolytes (DEEs) for zinc batteries was used to generate new electrolyte compositions. This database primarily focused on DEEs applied to zinc batteries, including all publications in this emerging field before the end of January 2024. Both review papers and experimental studies were included to ensure a broad and detailed understanding of the subject. To provide a broader context to the MAN, the database was supplemented with key papers on aqueous and hybrid electrolytes for zinc batteries, as well as seminal works on DEEs and zinc electrodeposition analysis. A full list of the papers used is provided in Table S1 (Supporting Information). To prepare the data for further analysis, the text of each paper was automatically extracted from the original PDF file and converted into a plain text format using the pdfminer.six Python package. Subsequently, key electrolyte composition details and performance metrics were extracted from each paper's plain text by generating short text summaries. This summarization process involved utilizing the gpt‐4‐0125‐preview model by OpenAI along with Prompt S1 (Supporting Information) through the LangChain Python library.^[^ ^32^, ^54^ ^]^ Each generated summary was saved as a separate text file for later integration into a vector database for retrieval‐augmented generation (RAG). The details of the process used to prepare each vector database and tune the hyperparameters for RAG can be found in the Supporting Information.

### Multi‐Agent Network Design

The multi‐agent network was implemented using LangChain libraries, specifically LangChain and Langgraph.^[^ ^32^, ^33^ ^]^ The two agents utilized OpenAI's gpt‐4‐0125‐preview model. Through initial parameter tuning, a model temperature of 0.5 was determined to provide the optimal trade‐off between fostering creative outputs and ensuring factual grounding. These agents were designed to work together, mimicking a collaborative research team.

A Chat Manager coordinated information flow and mediated agent access to RAG tools. The first agent, designated “Principal Investigator” (PI) and configured via Prompt S2 (Supporting Information), utilized a similarity search retriever to access a vector database of paper summaries, providing a broad overview of the DEE literature. The second agent, termed “Scientist” (Prompt S3, Supporting Information), accessed a chunked vector database of the full academic papers through an analogous retriever, enabling detailed factual verification and concept refinement. The paper and summary retrievers returned the top 4 paper chunks and summaries with the highest cosine similarity to the input retrieval query. This configuration mirrors a real‐world research team structure, where a PI typically possesses a broader perspective of the field, while a specialist focuses on specific details.

Composition generation followed an iterative workflow. For each candidate composition, the PI agent would analyze the literature summaries to identify promising directions, engaging in detailed discussion with the Scientist agent to refine and validate proposals, with the chat manager relaying messages between them. Once the agents had reached a conclusion, one of the agents would update the chat manager's state to “FINAL_ANSWER”, returning the proposed composition to the user.

This collaborative process was guided by two complementary approaches: an open‐ended exploration that encouraged broad innovation (Prompt S4, Supporting Information), followed by a focused investigation of zinc tetrafluoroborate hydrate‐based systems (Prompt S5, Supporting Information). The latter approach was chosen to systematically explore variations of this salt system while leveraging its known stability characteristics in DEE applications. An overall schematic diagram of the full multi‐agent workflow from initial composition generation through experimental validation is presented in Figure S20 (Supporting Information).

### Density Functional Theory Calculations

Density functional theory calculations were performed with Gaussian 09 at the B3LYP functional level of theory and a 6–31G(d,p) basis set. Molecular structures were initially drawn using Avogadro (1.2.0) and then geometrically optimized until the forces converged within a maximum of 4.5  ×  10^−4^ Hartrees Bohr^−1^ and root‐mean squared force of 3.0  ×  10^−4^ Hartrees Bohr^−1^.

The binding energies (*E_b_*) of each solvation structure were calculated using the following equation:

(1) $$E_{b}=E_{sys}\;-\sum_{i=1}^{n}E_{i}$$

where *E_sys_* is the energy of the optimized system, and *E_i_* is the energy of an individual component (i.e., H_2_O, Zn^2+^, BF_4_^−^, EC) after geometric optimization.

### Molecular Dynamics Simulations

Molecular dynamics simulations were performed with GROMACS using the OPLS‐AA force field.^[^ ^55^, ^56^ ^]^ The molecular topology for EC was generated using LigParGen, with restrained electrostatic potential (RESP) charges calculated using MultiWFN.^[^ ^57^, ^58^ ^]^ The topology for the BF_4_^−^ ion was taken from Doherty et al., and the TIP3P model was used to model H_2_O.^[^ ^59^ ^]^ For each ZE‐x system simulation, 100 Zn^2+^, 200 BF_4_^−^ ions, 565 water molecules, and the appropriate number of EC molecules were added to an 8 × 8 × 8 nm box using Packmol.^[^ ^60^ ^]^ 300, 600, 900, and 1200 molecules of EC were used for the ZE‐3, ZE‐6, ZE‐9, and ZE‐12 representative simulations, respectively. For the 2 m ZBF simulation, 100 Zn^2+^, 200 BF_4_^−^ ions, and 3343 water molecules were used. Long‐range electrostatic interactions were calculated using the Particle‐Mesh Ewald method with a Verlet cutoff scheme and a cutoff of 1.2 nm.^[^ ^61^ ^]^

The system was relaxed for 1 ns in a canonical ensemble, followed by 1 ns of relaxation in an isothermal‐isobaric ensemble. After NPT relaxation, the ZE‐3, ZE‐6, ZE‐9, and ZE‐12 systems converged to reasonable densities of 1.55, 1.45, 1.41, and 1.38 g cm^−3^, respectively. Finally, 5 ns of production was performed under isothermal‐isobaric ensemble conditions. A timestep of 1 fs was used in each case. Temperature was maintained at 296.15 K using a stochastic velocity rescaling (V‐rescale) thermostat with a time constant of 0.1 ps, and a stochastic cell rescaling (C‐rescale) barostat with a time constant of 1.0 ps maintained pressure at 1 bar.^[^ ^62^, ^63^ ^]^ Molecular dynamics snapshots were taken using VMD.^[^ ^64^ ^]^

The radial distribution function (*g*(*r*)) of each molecule surrounding the Zn ion was calculated using the expression:

(2) $$g\;\left(r\right)=\frac{n_{r}}{4\pi r^{2}\rho \Delta r}$$

The distance of a species from a reference atom (in this case, Zn) is defined by *r*, ρ represents the mean probability density of the species in the electrolyte, and *n_r_* denotes the number of particles present in a layer with a thickness of Δ*r*. The carbonyl group oxygen in EC, the oxygen atom in water, and one of the fluorine atoms in BF_4_^−^ were chosen as the reference positions for calculating g(r). The cumulative number rdf, *N*(*r*), was then calculated by integrating over the desired interval using the following equation:

(3) $$N\;\left(r\right)=\int_{0}^{r}4\pi \rho r^{2}g\left(r^{\prime }\right)dr^{\prime }$$

The mean squared displacement (MSD) of a species was calculated using the equation:

(4) $$MSD\;\left(t\right)=\frac{1}{N}\;\sum_{i=1}^{N}\left|r_{i}\left(t\right)-r_{i}\left(t_{0}\right)\right|$$

where N represents the total number of species in the tested system, and *r_i_* (*t*) and *r_i_* (*t*_0_) are the positions of a given species at time *t* and *t*_0_ =  0, respectively.

### Electrolyte Preparation

Zinc tetrafluoroborate hydrate (ZBF, Aladdin, ≥18 wt% Zn), meso‐erythritol (ERY, Aladdin, 99%), glycerol (GLY, Sigma–Aldrich, ≥99%), ethylene carbonate (EC, Sigma Aldrich, >99%), levulinic acid (LEV, TCI, >97%) and lactic acid (LAC, TCI, >95%) were purchased from their respective suppliers and used without further processing. The molecular formula of ZBF was found to be Zn(BF_4_)_2_·5.65H_2_O by thermogravimetric analysis. The method and results used to obtain this value are presented in Supporting Information.

To prepare each electrolyte, 0.5 g of ZBF was weighed into a vial using a Mettler Toledo XS105 microbalance. The appropriate masses of each Lewis base were then weighed into the vial to obtain the desired molar ratio. The electrolyte systems containing levulinic acid, lactic acid, ethylene carbonate, and glycerol were stirred at 50 °C for 2 h. The system containing erythritol was heated initially to 130 °C for 15 min while stirring to melt the Lewis base. The temperature was then reduced to 80 °C for the remainder of the 2 h.

### Cathode Preparation

PANI cathodes were produced by mixing commercially available PANI (Yuanda Materials LLC) with super P conductive carbon and a Daikin D210c PTFE emulsion, such that the mass ratio of PANI:carbon:PTFE was 65:25:10. The mixture was then mechanically homogenized and rolled into a thin sheet before pressing into a 316‐grade SS mesh. The finished cathodes were then dried overnight in a vacuum oven at 70 °C. The mass loading of PANI in the cathodes was between 1.8 and 2.1 mg cm^−2^, unless otherwise stated.

### Electrochemical Testing

EIS and linear sweep voltammetry characterizations were performed on a Biologic VSP‐300. Electrolyte conductivity was measured by determining the resistance of SS symmetric cells (SS||SS) using EIS. The electrolyte conductivity, σ, was then calculated using the equation:

(5) $$\sigma =\frac{L}{RA}$$

where *R* is the resistance of the cell, *A* is the area of the SS electrode, and *L* is the thickness of the fiberglass separator.

### Cell Preparation

SS||SS, Zn||Zn, Zn||SS and Zn||PANI cells were assembled into stainless steel CR2023 coin cell casings in air. Zn electrodes with 50 µm thickness and 12 mm diameter were used for the Zn||Zn and Zn||SS cells, while a larger 16 mm diameter Zn anode was used in the Zn||PANI full cells. Glass fiber filters (Whatman GF/A), with a nominal thickness of 260 µm, were used as separators in all cells, along with 120 µl of electrolyte.

### Materials Characterizations

DSC was performed using a TA Instruments Discovery DSC 2500 with a cooling rate of 5 °C min^−1^ under N_2_ flow. X‐ray diffraction was performed with a Malvern Panalytical X'Pert Pro X‐ray diffractometer with Cu‐Kα (1.5406 Å wavelength) radiation. A Bruker Vertex 70 was used for Fourier transform infrared spectroscopy measurements, which were collected between 4000–400 cm^−1^. Cycled Zn anode surfaces were imaged with a JEOL JSM‐6390 SEM with EDX spectra captured using an Oxford Instruments Silicon Drift Detector. XPS measurements were performed with an AXIS Supra+. Zn anodes were thoroughly washed in water and ethanol, then vacuum dried at room temperature prior to FTIR and XPS analysis.

## Conflict of Interest

The authors declare no conflict of interest.

## Supporting information



Supporting Information

## Data Availability

The data that support the findings of this study are available from the corresponding author upon reasonable request. The software developed for this work is openly available at: https://github.com/mjrobson/multi‐agent‐electrolyte‐discovery.
