# Supplementary material for: Multi‐Agent‐Network‐Based Idea Generator for Zinc‐Ion Battery Electrolyte Discovery: A Case Study on Zinc Tetrafluoroborate Hydrate‐Based Deep Eutectic Electrolytes
Source: Adv Mater. 2025 May 22;37(32):2502649. doi: 10.1002/adma.202502649 (PMC12355564; doi:10.1002/adma.202502649)
Supplement: Supplementary file 1 — Supporting Information [file ADMA-37-2502649-s001.pdf]

ADVANCED MATERIALS

Supporting Information

for *Adv. Mater.*, DOI 10.1002/adma.202502649

Multi-Agent-Network-Based Idea Generator for Zinc-Ion Battery Electrolyte Discovery: A Case Study on Zinc Tetrafluoroborate Hydrate-Based Deep Eutectic Electrolytes

*Matthew J. Robson, Shengjun Xu, Zilong Wang, Qing Chen and Francesco Ciucci**

Supporting Information

for

Multi-agent-network-based idea generator for zinc-ion battery electrolyte discovery: A case study on zinc tetrafluoroborate hydrate-based deep eutectic electrolytes.

Matthew J. Robson[a], Shengjun Xu[b,c], Zilong Wang[a], Qing Chen[a,d,e], Francesco Ciucci[a,b,c].*

a. Department of Mechanical and Aerospace Engineering, The Hong Kong University of Science and Technology, Hong Kong SAR, China.
b. University of Bayreuth, Chair of Electrode Design for Electrochemical Energy Systems, 95448 Bayreuth, Germany.
c. University of Bayreuth, Bavarian Center for Battery Technology (BayBatt), Universitätsstraße 30, 95447 Bayreuth, Germany.
d. The Energy Institute, The Hong Kong University of Science and Technology, Clear Water Bay, Kowloon, Hong Kong SAR, China.
e. Department of Chemistry, The Hong Kong University of Science and Technology, Clear Water Bay, Kowloon, Hong Kong SAR, China.

*E-mail: francesco.ciucci@uni-bayreuth.de

Table of Contents

Supplementary Experimental Methods 3

Retrieval augmented generation and hyperparameter tuning 3

Determining the hydration level of $Zn(BF_4)_2 \cdot xH_2O$ 5

Supplementary Prompts 10

Supplementary Figures 15

Supplementary Tables 35

References 44

Supplementary Experimental Methods

Retrieval augmented generation and hyperparameter tuning

Optimization of Retrieval Augmented Generation (RAG) hyperparameters is crucial for achieving optimal RAG performance. Vector databases were constructed to store summaries and full-text papers. These databases leveraged the tiktoken byte pair encoder and were built using ChromaDB and OpenAI embeddings. Each summary was stored as a single chunk to preserve all contextual details, ensuring that all summaries were passed to the multi-agent network (MAN) during retrieval. For the paper database, given the longer context length, an initial optimization of the tiktoken encoder's chunk size and overlap size was performed using the Retrieval Augmented Generation Assessment (Ragas) package[1]. Response answer correctness was used as the primary metric for this evaluation. To calculate this parameter, a subset of the data containing 10 randomly chosen papers was selected. Five questions were formulated based on the information explicitly stated within these papers. Each question was then presented to GPT-4 along with the relevant portion of the text for ground-truth response generation, and the accuracy of the generated responses was manually confirmed. A retrieval-augmented question-answering (QA) approach was then employed to quantify RAG performance. This approach involved utilizing a separate GPT-4 instance for answering each question, along with access to the database through a similarity search retriever. The generated response, the ground-truth response, and the retrieved RAG text documents were then fed into the GPT-3.5-turbo model to determine the number of true positive, false positive, and false negative statements. The answer correctness was then quantified using the following equation:

$$\text{answer correctness} = \frac{|TP|}{(|TP| + 0.5 \times (|FP| + |FN|)} \tag{1}$$

where TP is the number of true positive statements (statements which are present in both the ground truth answer and the generated answer), FP is the number of false positive statements (statements present in the answer but not in the ground truth), and FN is the number of false negative statements (not in the answer but present in the ground truth) within the output response. Following optimization, an optimal chunk size of 2000 tokens with an overlap of 100 was selected for the full paper database used in the final model (**Figure S1**).

Determining the hydration level of $Zn(BF_4)_2{\cdot}xH_2O$

The molar mass of $Zn(BF_4)_2{\cdot}xH_2O$ (ZBF) was determined using the method outlined by Han et al.[2] A sample of ZBF was heated from room temperature to 400 °C with a 2 °C/min heating rate under N_2 flow, using a TA instrument Q5000 thermogravimetric analyzer with the results presented in **Figure S3**. Initially, water began to evaporate from the ZBF, followed by the decomposition of $Zn(BF_4)_2$ into ZnF_2 and further water loss via the reaction intermediate of Zn(OH)F. At the end of the testing period, only ZnF_2 remained, as confirmed by XRD (**Figure S3b**). The mole fraction of water was then determined using the ratio of the initial mass of ZBF to the final mass of ZnF_2 at the end of the TGA process using the following equation:

$$x = \frac{M_{\mathrm{ZnF_2}} m_{\mathrm{Zn(BF_4)_2 \cdot xH_2O}} - M_{\mathrm{Zn(BF_4)_2}} m_{\mathrm{ZnF_2}}}{M_{\mathrm{H_2O}} m_{Zn\mathrm{F_2}}} \tag{6}$$

Where M_a represents the molar mass of component a in g/mol and m_a is the mass of component a in g. Using the above equation, the composition was found to be $Zn(BF_4)_2{\cdot}5.65H_2O$

Multi-agent network example agent transcript

The following excerpt from the MAN dialogue illustrates its reasoning process when proposing EC as an electrolyte component. The system initially recommended a ZBF-glycerol-lactic acid composition based on prior knowledge. Following the Scientist's additional literature search, a ZBF-ethylene glycol electrolyte was identified in the literature.[2] The MAN subsequently proposed EC as a novel Lewis base alternative by combining literature-derived evidence of BF_4^-'s interfacial stabilization with the LLM's pre-trained knowledge of EC's dielectric and thermal properties. This sequence exemplifies how the RAG-augmented decision pathway supplies critical context, steering composition generation toward chemically relevant alternative compositions.

Agent dialogue excerpt demonstrating the MAN's selection rationale for EC in ZBF-based electrolytes

User Prompt: Create a highly novel composition for a hydrated deep eutectic electrolyte based on zinc tetrafluoroborate hydrate that could be used in Zinc batteries...

Function call to Summary Retriever: Query("Zinc tetrafluoroborate hydrate deep eutectic electrolyte")

Principal Investigator: Based on the information provided, it's clear that the field of deep eutectic solvent (DES) electrolytes for zinc batteries is quite developed, with a variety of compositions tested, including those involving choline chloride, ethylene glycol, urea, and various zinc salts. However, zinc tetrafluoroborate hydrate has not been mentioned in the summaries provided, suggesting an opportunity for novelty in this area.
Given the goal of proposing a highly novel hydrated deep eutectic electrolyte composition… I propose the following composition:
Novel Electrolyte Composition

1. **Zinc Tetrafluoroborate Hydrate ($Zn(BF_4)_2 \cdot xH_2O$):** Acts as the source of zinc ions for ionic conduction. Its hydration enhances the ionic conductivity and stability of the electrolyte.
2. **Glycerol ($C_3H_8O_3$):** A widely used and environmentally friendly polyol that can serve as a hydrogen bond donor in the formation of a deep eutectic solvent…
3. **Lactic Acid ($C_3H_6O_3$):** A naturally occurring organic acid that can act as a complexing agent for zinc ions and a hydrogen bond donor in the DES. [...]

Rationale

…This composition is expected to be novel due to the use of zinc tetrafluoroborate hydrate in combination with glycerol and lactic acid, which has not been reported in the provided summaries…

Next Steps for Validation

- **Novelty Check:** A more comprehensive literature review should be conducted to ensure that this specific combination has not been previously explored…
  This proposal is open for further refinement and approval by the principal investigator before proceeding to experimental testing.

Scientist: [Initiates targeted literature search]

Function call to Paper Retriever: Query("Zinc tetrafluoroborate hydrate")

Function Message from Paper Retriever:
Excerpt from retrieved text (Han et al., Nature Sustainability, 2022):
"Here, we report a non-flammable hydrous organic electrolyte combining a hydrated $Zn(BF_4)_2$ salt with an ethylene glycol (EG) solvent for sustainable Zn batteries. The $Zn(BF_4)_2$/EG electrolyte not only promotes the in situ formation of a ZnF_2 SEI to suppress Zn dendrite growth and side reactions but also features extremely low volatility and a wide operating temperature range. As a result, the Zn electrode exhibits a long-term cycling stability of over 4,000 h at 0.5 mA cm^{-2}, a high Coulombic efficiency (CE) of 99.4%, a large areal capacity of 5 mAh cm^{-2} with a high depth of discharge (DOD) of 50% and wide operating temperature range (−30 °C to 40 °C)… The current non-flammable $Zn(BF_4)_2$/EG electrolyte opens a pathway towards safe, cost competitive and high-performance electrolyte design for practical and sustainable Zn batteries."…

Scientist: Based on the detailed information about the use of zinc tetrafluoroborate hydrate ($Zn(BF_4)_2 \cdot xH_2O$) in a non-flammable hydrous organic electrolyte for zinc batteries, it appears that this composition has already been explored and demonstrated significant advantages in terms of safety, performance, and sustainability. The $Zn(BF_4)_2$/EG (ethylene glycol) electrolyte promotes the in situ formation of a ZnF_2 solid-electrolyte interphase (SEI) to suppress dendrite growth and side reactions, features extremely low volatility, and operates over a wide temperature range, contributing to long-term cycling stability, high Coulombic efficiency, and improved capacity retention.
Given this context, to propose a novel hydrated deep eutectic electrolyte composition that includes $Zn(BF_4)_2 \cdot xH_2O$ and improves upon the existing $Zn(BF_4)_2$/EG system while excluding choline chloride, we might consider integrating insights from the latest research and exploring novel combinations that could potentially enhance the electrolyte's performance, safety, or environmental friendliness…

Revised Novel Electrolyte Composition Proposal:

1. **Zinc Tetrafluoroborate Hydrate ($Zn(BF_4)_2 \cdot xH_2O$):** Maintains its role as the source of zinc ions for ionic conduction, leveraging the benefits of its hydration for enhanced ionic conductivity and stability.
2. **Ethylene Carbonate (EC):** A common solvent in lithium-ion batteries, ethylene carbonate could be explored as a component of the deep eutectic solvent system for zinc batteries. EC has a high dielectric constant, which can increase the electrolyte's ability to dissolve salts and potentially enhance ionic conductivity. It

also has a high boiling point, contributing to the thermal stability of the electrolyte.

3. **Lithium Bis(trifluoromethanesulfonyl)imide (LiTFSI):** Although traditionally used in lithium-ion batteries, LiTFSI could be considered for its role in enhancing the solvation environment of $Zn(BF_4)_2 \cdot xH_2O$, potentially improving the zinc ion transport and suppressing dendrite formation. LiTFSI is known for its wide electrochemical stability window and could contribute to the formation of a stable SEI layer on the zinc anode.

Rationale for the Revised Composition:

- **Ethylene Carbonate:** The inclusion of EC is aimed at leveraging its high dielectric constant and thermal stability to enhance the electrolyte's performance and safety.
- **LiTFSI:** Its addition is intended to improve the solvation dynamics and electrochemical stability of the electrolyte, potentially enhancing zinc ion mobility and contributing to dendrite suppression.

Next Steps for Validation:

- **Novelty and Compatibility Check:** Conduct a thorough literature review and experimental testing to confirm the novelty and compatibility of the proposed components with $Zn(BF_4)_2 \cdot xH_2O$ in a deep eutectic solvent system.
- **Performance Evaluation:** Synthesize the proposed electrolyte and evaluate its ionic conductivity, electrochemical stability window, dendrite suppression capabilities, and overall battery performance.
- **Safety and Environmental Impact Assessment:** Assess the safety profile and environmental impact of the proposed electrolyte, considering the toxicity and biodegradability of its components.

This revised proposal aims to explore uncharted territories in the composition of hydrated deep eutectic electrolytes for zinc batteries, with a focus on enhancing performance, safety, and sustainability. Approval from the principal investigator would be required before proceeding with experimental validation.

Principal Investigator: Based on the detailed information about the use of zinc tetrafluoroborate hydrate ($Zn(BF_4)_2 \cdot xH_2O$) in a non-flammable hydrous organic electrolyte for zinc batteries, it appears that this composition has already been explored and demonstrated significant advantages in terms of safety, performance, and sustainability. The $Zn(BF_4)_2$/EG (ethylene glycol) electrolyte promotes the in situ formation of a ZnF_2 solid-electrolyte interphase (SEI) to suppress dendrite growth and side reactions, features extremely low volatility, and operates over a wide temperature range, contributing to long-term cycling stability, high Coulombic efficiency, and improved capacity retention.

Given this context, to propose a novel hydrated deep eutectic electrolyte composition that includes $Zn(BF_4)_2 \cdot xH_2O$ and improves upon the existing $Zn(BF_4)_2$/EG system…

Revised Novel Electrolyte Composition Proposal:

1. **Zinc Tetrafluoroborate Hydrate ($Zn(BF_4)_2 \cdot xH_2O$):** Maintains its role as the source of zinc ions for ionic conduction, leveraging the benefits of its hydration for enhanced ionic conductivity and stability.
2. **Ethylene Carbonate (EC):** A common solvent in lithium-ion batteries, ethylene carbonate could be explored as a component of the deep eutectic solvent system for zinc batteries. EC has a high dielectric constant, which can increase the electrolyte's ability to dissolve salts and potentially enhance ionic conductivity. It

also has a high boiling point, contributing to the thermal stability of the electrolyte.

3. **Lithium Bis(trifluoromethanesulfonyl)imide (LiTFSI):** Although traditionally used in lithium-ion batteries, LiTFSI could be considered for its role in enhancing the solvation environment of $Zn(BF_4)_2 \cdot xH_2O$, potentially improving the zinc ion transport and suppressing dendrite formation. LiTFSI is known for its wide electrochemical stability window and could contribute to the formation of a stable SEI layer on the zinc anode.

Rationale for the Revised Composition:

- **Ethylene Carbonate:** The inclusion of EC is aimed at leveraging its high dielectric constant and thermal stability to enhance the electrolyte's performance and safety.
- **LiTFSI:** Its addition is intended to improve the solvation dynamics and electrochemical stability of the electrolyte, potentially enhancing zinc ion mobility and contributing to dendrite suppression…

Supplementary Prompts

Prompt S1. Prompt passed to GPT-4 to summarize each of the papers in the database. The {paper} variable is replaced with the extracted raw text from each paper as a string.

You are an expert researcher in the field of electrochemistry. Your job is to concisely summarize the academic paper from the field of deep eutectic electrolytes (DEEs) (delimited by triple backticks) following the framework given below:
In particular, please identify the following information from the paper:

1) Electrolyte composition: Please identify all electrolyte components used and their relative proportions in the electrolyte. When you identify an electrolyte component by whatever means, refer to it in the summary with the full name, abbreviation and any other common names given to the compound and its chemical formula. For example, SL would be replaced with Sulfolane (SL, tetramethylene sulfone, (CH2)4SO2)
2) Cathode composition: Please identify the composition of cathode used in the battery. If a full cell is not tested, please respond with "Full cell not tested." for this point.
3) Performance parameters: Please summarize the relevant performance parameters for the electrolyte here. You should include the electrolyte ionic conductivity, Electrochemical window, and relevant symmetric cell cycling information. Full battery performance such as specific capacity, given in mAh/g should also be added, where available.
4) Rationale: Please briefly summarize the rationale given in the paper for the success of the chosen composition.

An example output is provided for you below:
"Title: Hydrated Deep Eutectic Electrolytes for High-Performance Zn-Ion Batteries Capable of Low-Temperature Operation
1) Electrolyte composition: Zinc perchlorate hexahydrate ($Zn(ClO_4)_2 \cdot 6H_2O$) + 85 mol% Sulfolane (SL, tetramethylene sulfone, (CH2)4SO2)
2) Cathode composition: Polyaniline (PANI) + Ketjenblack (C) with a polytetrafluoroethylene (PTFE, Teflon) binder in a mass ratio of 8:1:1. The mass loading of the cathode was 2 mg/cm^2
3) Performance Parameters: Electrolyte conductivity 2.0 mS/cm. The electrolyte showed stable cycling for 800 h @ 0.5 mA/cm^2 and 0.5 mAh/cm^2 in a Zn|Zn symmetric cell. Stable cycling for 2000 h @ 0.2 mA/cm^2 and 0.1 mAh/cm^2 was also achieved in a similar cell. A Zn|DEE|PANI full cell showed a stable capacity of 71 mAh/g for 2500 cycles @ 3 A/g.
4) Rationale: The coordination between sulfolane and Zn^{2+} leads to an enhanced eutectic effect, broadening the DEE's operating temperature range. This special water-in-DEE structure improves ionic movement, aids Zn^{2+} deposition, and lessens water reactivity, as shown by simulations and tests. The DEE prevents dendrite growth, enabling Zn|DEE|Zn cells to operate for thousands of hours without short-circuiting."

```
{paper}
```

Prompt S2. Agent description given to the Principal Investigator agent.

```
You are an exceptionally creative and rigorous scientist. You create and approve new electrolyte compositions for deep eutectic electrolyte batteries, with the support of detailed information from the scientist. You ensure every electrolyte composition you propose is completely novel.
```

Prompt S3. Agent description given to the Scientist agent.

```
You are a highly intelligent and diligent scientist. You provide detailed
and accurate information to support the Principal Investigator using the
paper_retriever. When searching for detailed information about electrolyte
compositions you use all common names for each electrolyte component. For
example, instead of Sulfolane, you would search for Sulfolane (SL,
Tetramethyl Sulfone). To find detailed information for an electrolyte
component, you may wish to search for only one component per tool request.
You also provide criticism and suggestions to improve the electrolyte
compositions proposed by the Principal Investigator.
```

Prompt S4. First user prompt passed to the Principal Investigator used to initiate electrolyte composition generation. This prompt does not mention ZBF to enhance output creativity.

```
Create a completely novel composition for a hydrated deep eutectic electrolyte that could be used in Zinc batteries.
Check the costs and environmental impact and identify possible electrolyte compositions that improve on your suggestion. Modify the electrolyte composition to make improvements in battery performance, safety or environmental friendliness as appropriate. You may wish to consider lewis acids and lewis bases typically used in other fields, or natural deep eutectic electrolytes to add novelty."
Check the novelty of the final composition. Ensure that the final composition contains a hydrated zinc salt for ionic conduction.
The Principal Investigator must approve the final deep eutectic electrolyte composition for testing. Please return the final electrolyte composition and your logic for choosing it. Please propose a highly novel hydrated deep eutectic electrolyte with 3 or fewer components.
```

Prompt S5. Second user prompt passed to the Principal Investigator Agent to initiate composition generation.

```
Create a completely novel composition for a zinc tetrafluoroborate-based
hydrated deep eutectic electrolyte that could be used in Zinc batteries.
Check the costs and environmental impact and identify possible electrolyte
compositions that improve on your suggestion. Modify the electrolyte
composition to make improvements in battery performance, safety or
environmental friendliness as appropriate. You may wish to consider Lewis
acids and Lewis bases typically used in other fields, or natural deep
eutectic electrolytes to add novelty.
Check the novelty of the final composition. Ensure that the final
composition contains zinc tetrafluoroborate for ionic conduction.

The Principal Investigator must approve the final deep eutectic electrolyte
composition for testing. Please return the final electrolyte composition
and your logic for choosing it. Please propose a highly novel hydrated deep
eutectic electrolyte with 3 or fewer components.
```

Supplementary Figures

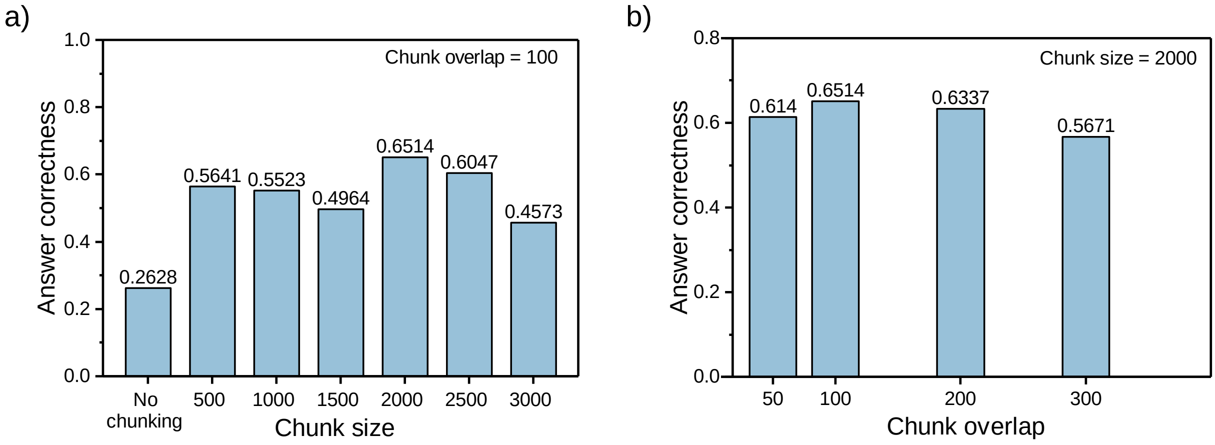

Figure S1. Plots of answer correctness from the RAG assessment process with different a) chunk size at a fixed chunk overlap of 100, and b) chunk overlap with a fixed chunk size of 2000.

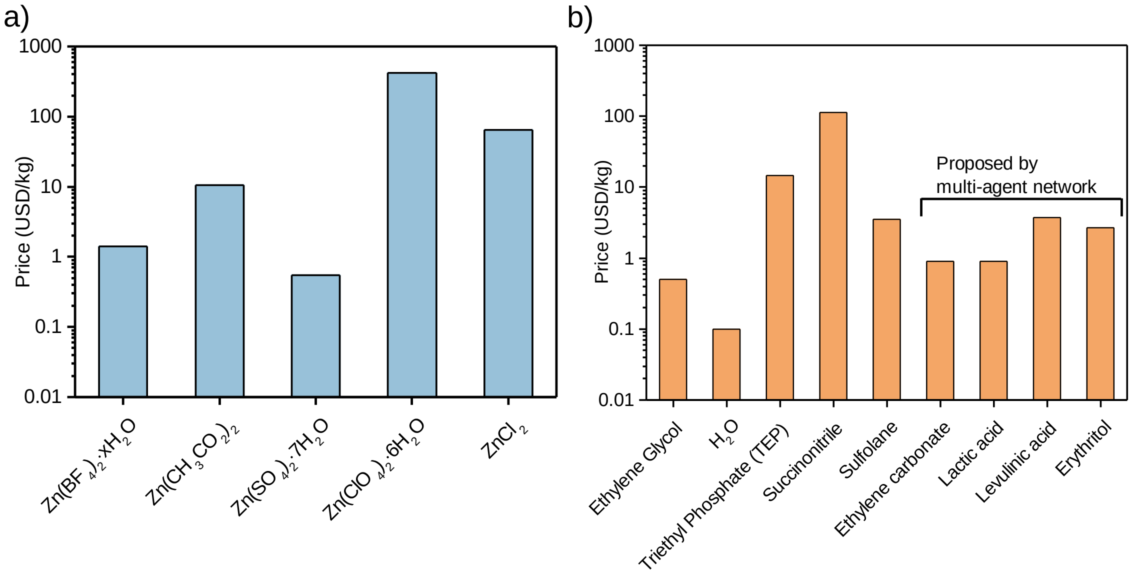

Figure S2. Cost comparison of a) ZBF against other common Lewis acids and b) the Lewis bases proposed by the multi-agent network against other common Lewis bases. The prices used in this work were obtained from [2] and from Alibaba.com, taken in May 2024.

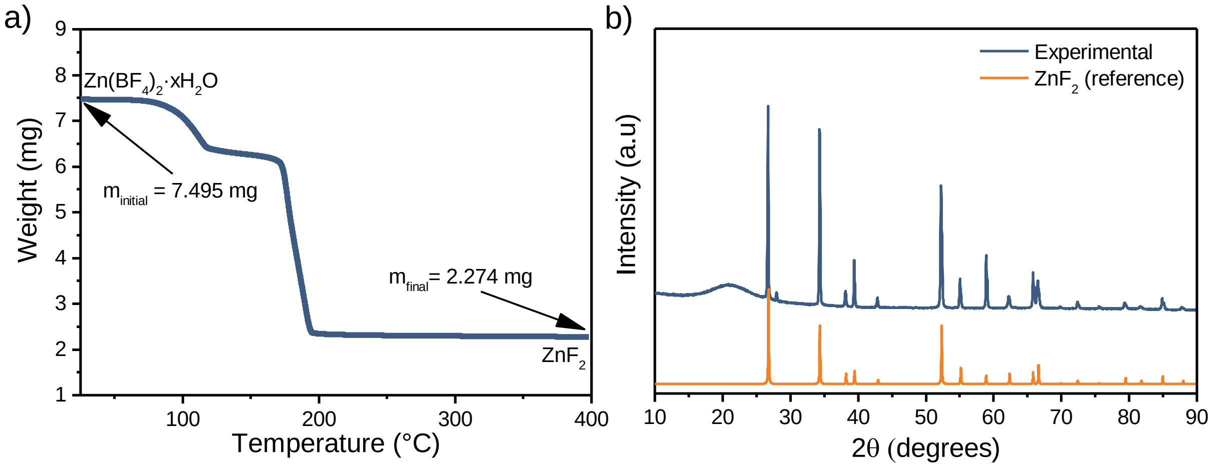

Figure S3. a) Plot of mass loss from TGA of ZBF, and b) XRD spectrum of the powder remaining at the end of TGA. A clear structural match between the calculated spectrum for ZnF_2 and the experimental spectrum is observed. The reference ZnF_2 spectrum was obtained from the Crystallography Open Database (number 9007539).

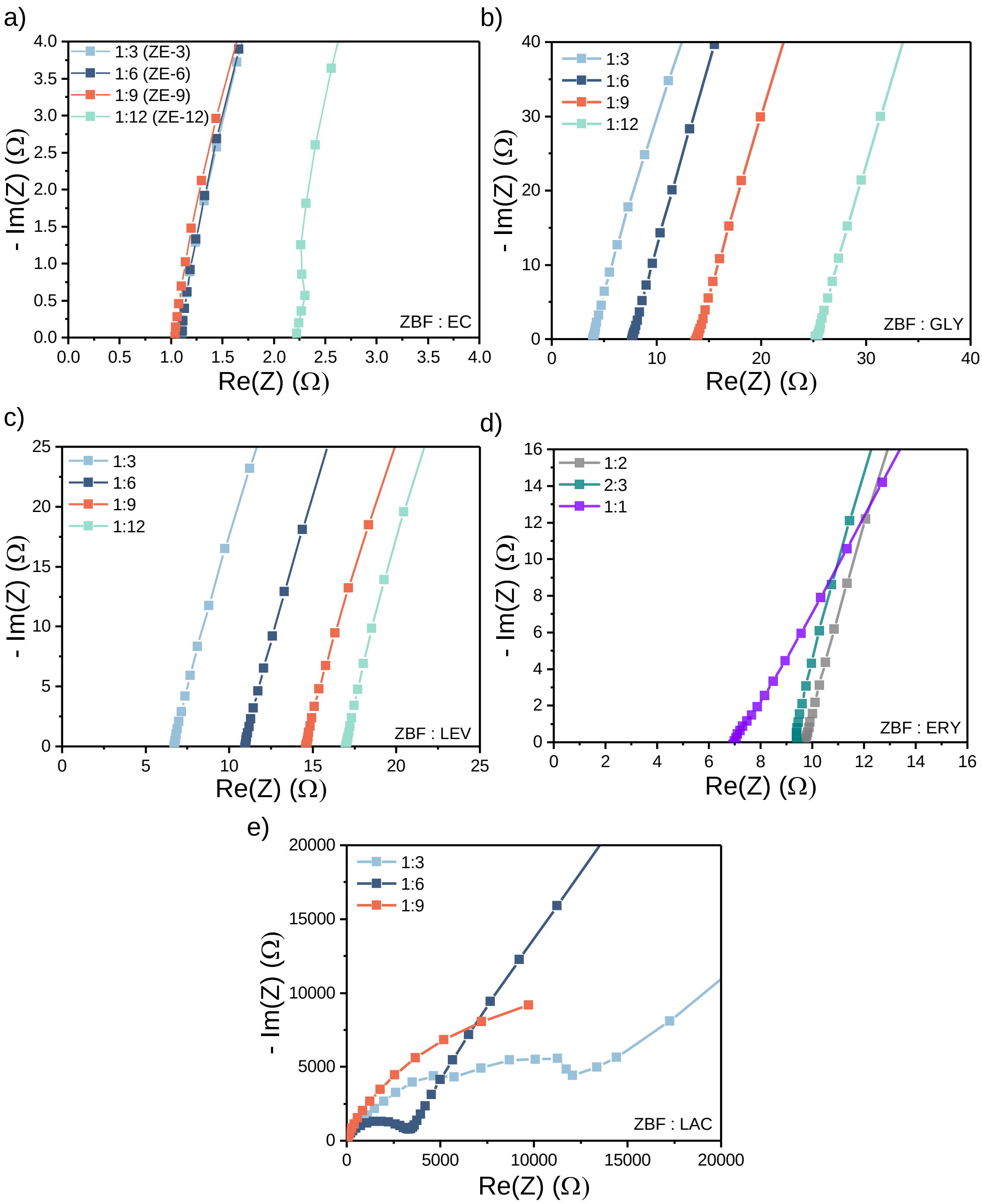

Figure S4. Electrochemical impedance spectra collected from SS||SS cells containing electrolyte systems based on ZBF paired with: a) ethylene carbonate (ZBF-EC, data shown for various ZE-x molar ratios, where x represents the molar ratio of EC to ZBF), b) glycerol (ZBF-GLY), c) levulinic acid (ZBF-LEV), d) erythritol (ZBF-ERY), and e) lactic acid (ZBF-LAC).

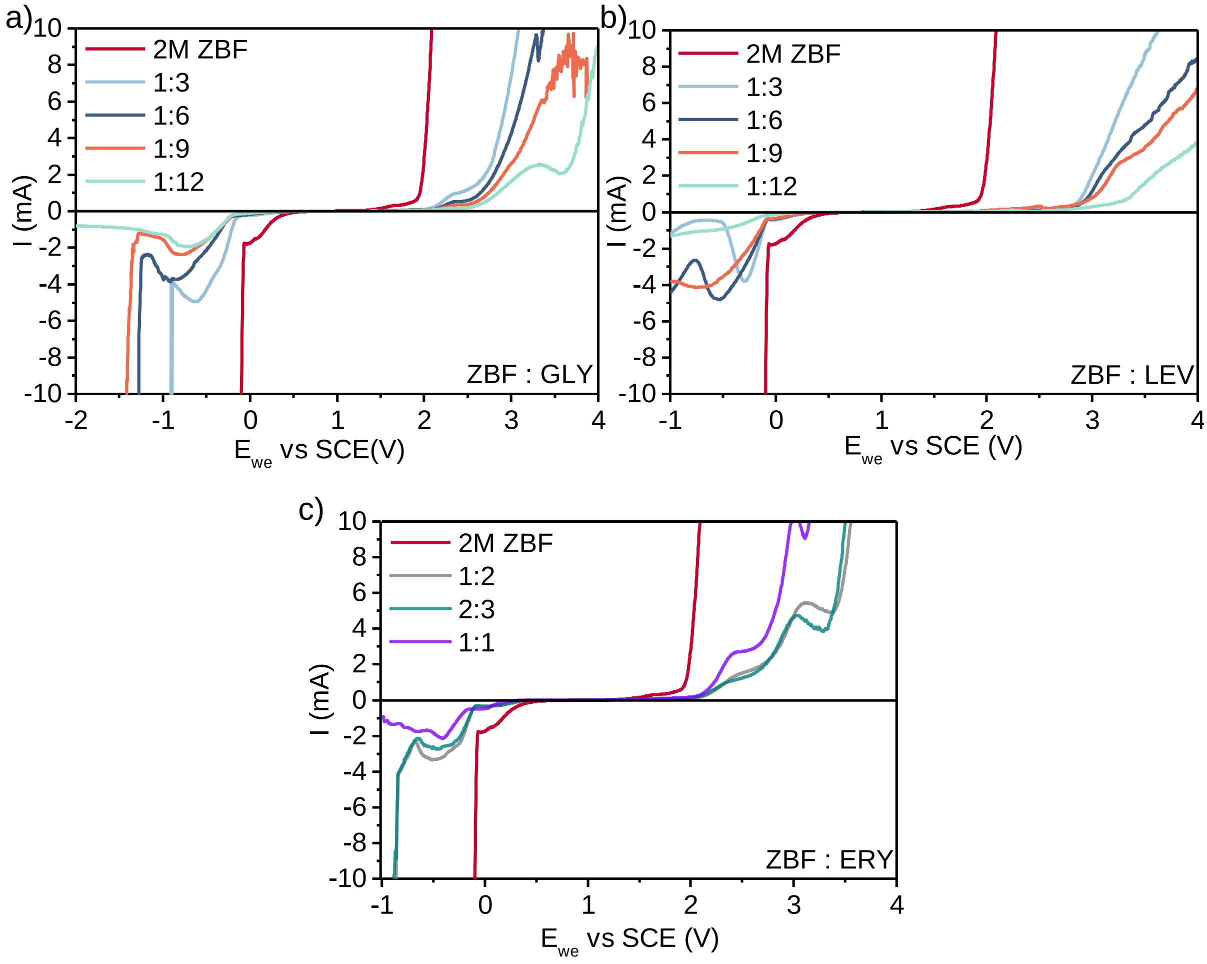

Figure S5. Linear sweep voltammograms from Zn|SS cells with a) ZBF-GLY, b) ZBF-LEV, c) ZBF-ERY electrolytes. All voltammograms were captured at a scan rate of 1 mV/s.

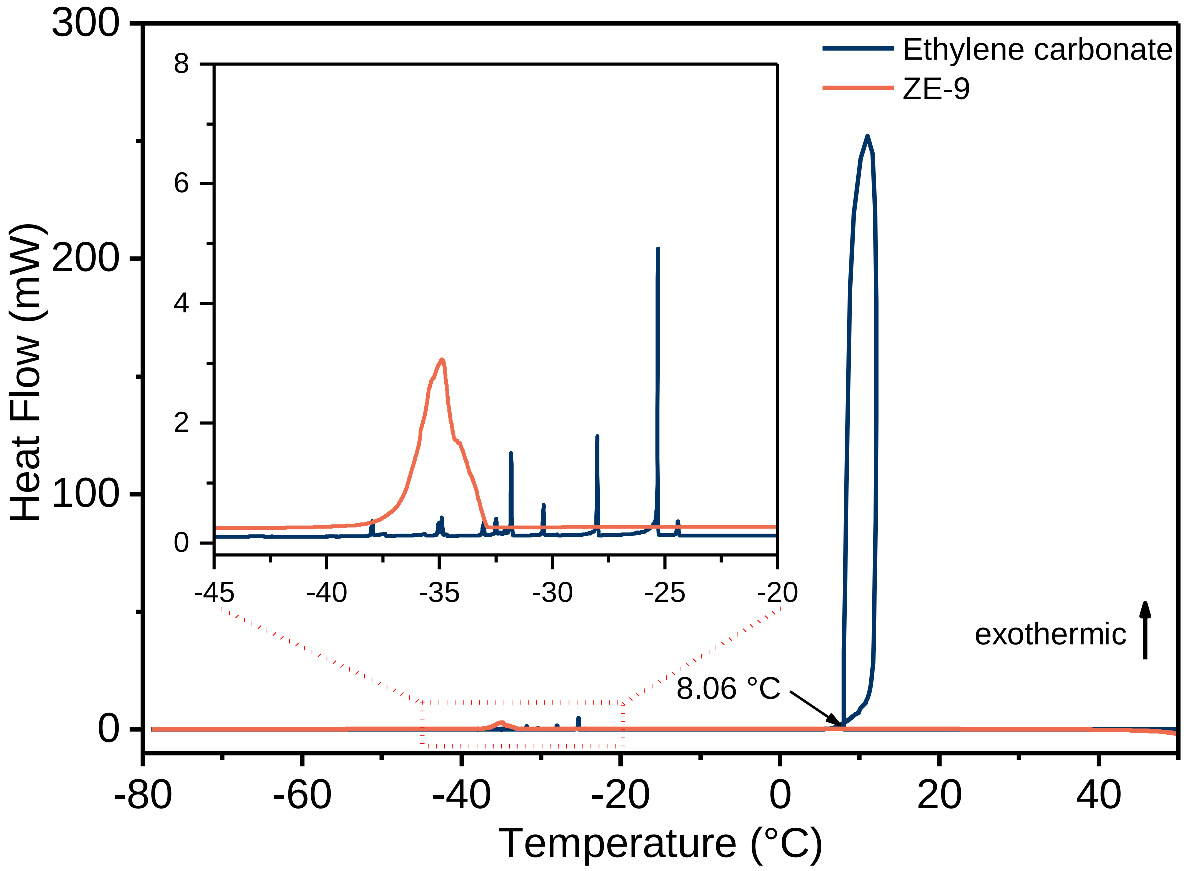

Figure S6. Heat flow as a function of temperature, derived from differential scanning calorimetry conducted on the ZE-9 electrolyte and a reference sample of pristine ethylene carbonate.

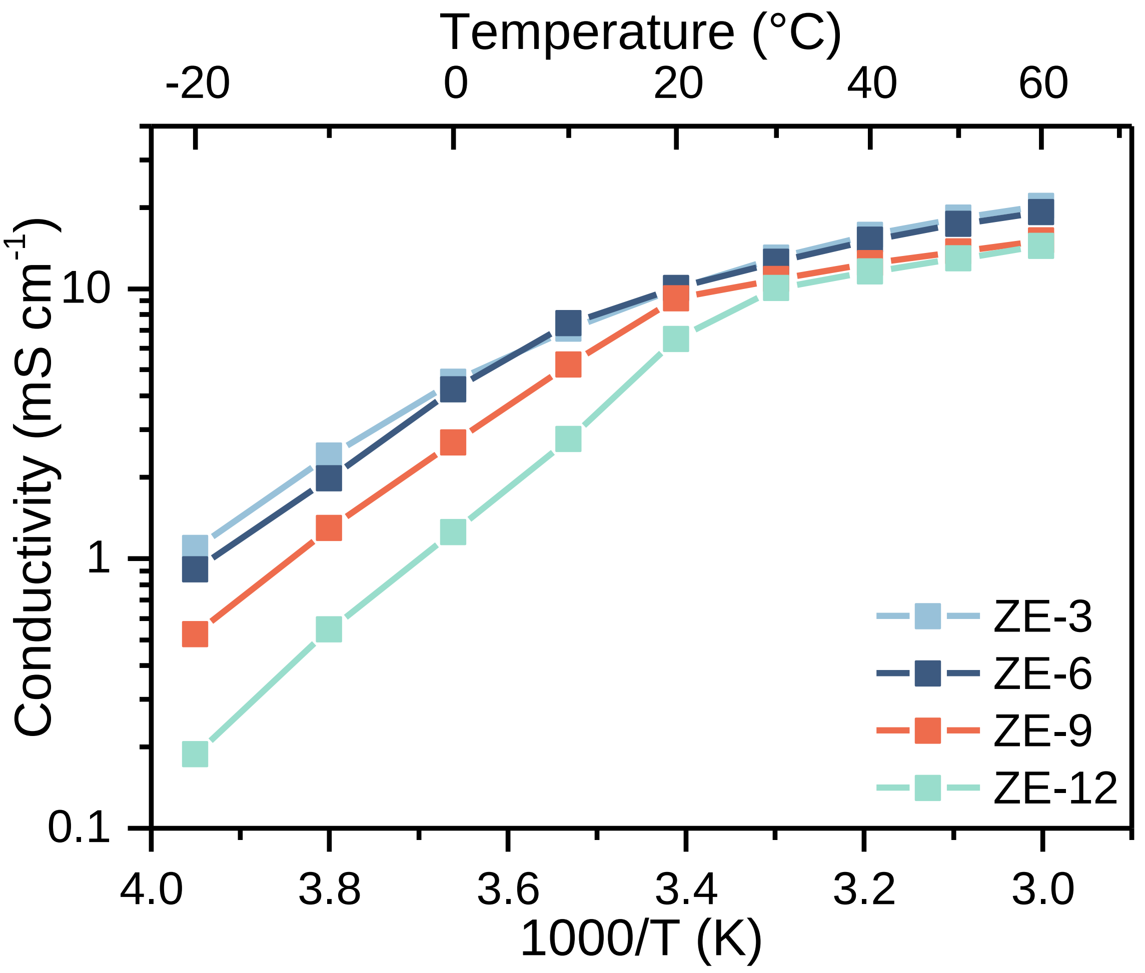

Figure S7. Conductivity against temperature for the ZE-x electrolytes.

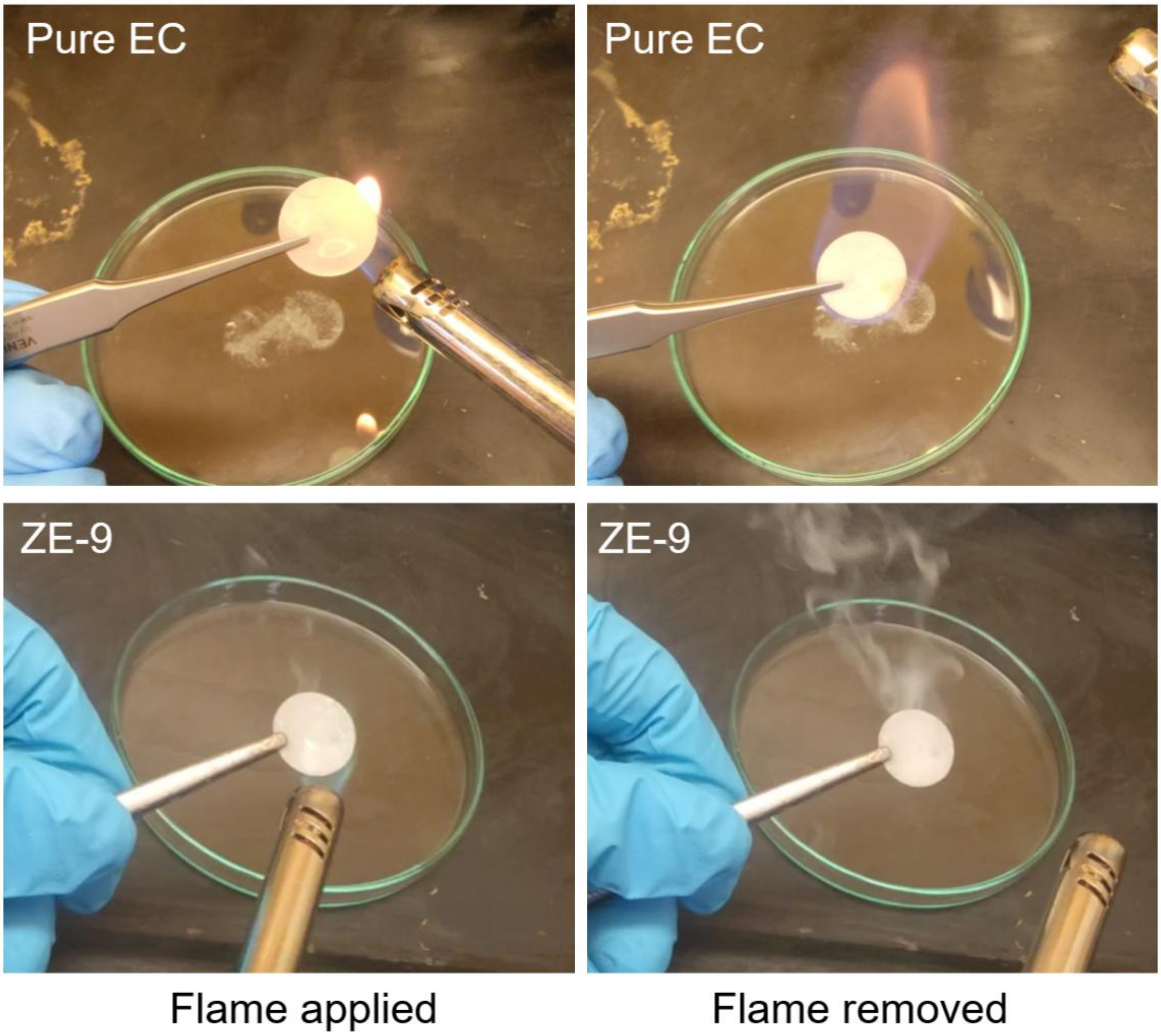

Figure S8: Still images flame exposure of fiberglass separators soaked in pure EC versus ZE-9 electrolyte. The ZE-9-soaked separator exhibits a brief blue-green flame that self-extinguishes upon removal of the ignition source. In contrast, the EC-soaked separator sustains combustion after ignition.

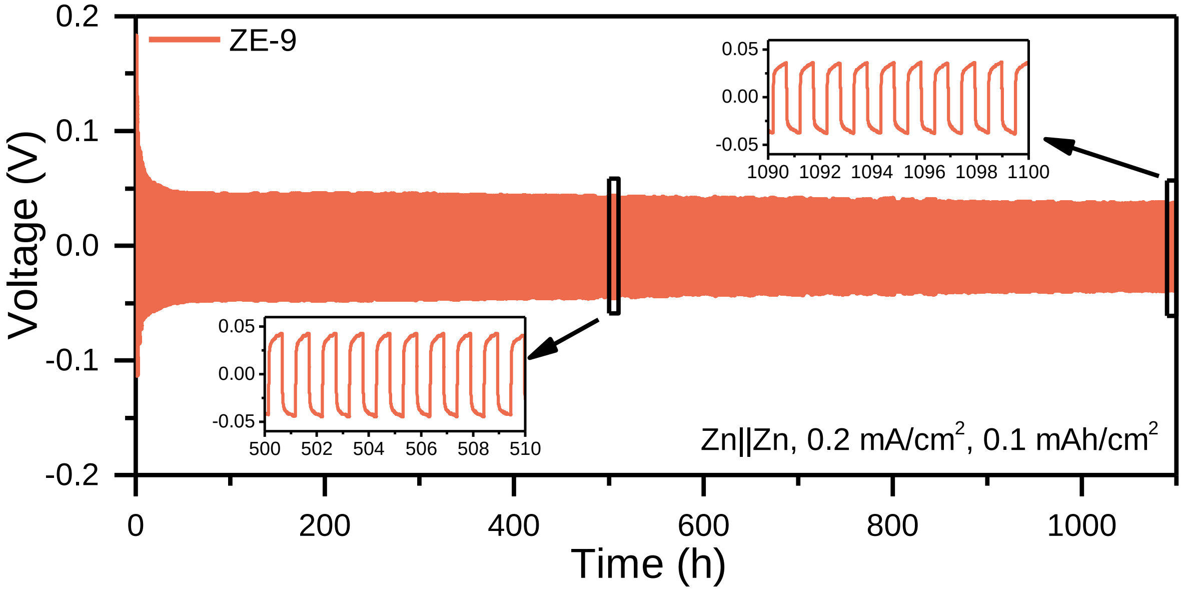

Figure S9. Voltage profiles from Zn|ZE-9|Zn symmetric cell cycling, collected at 0.2 mA cm^{-2} and 0.1 mAh cm^{-2}.

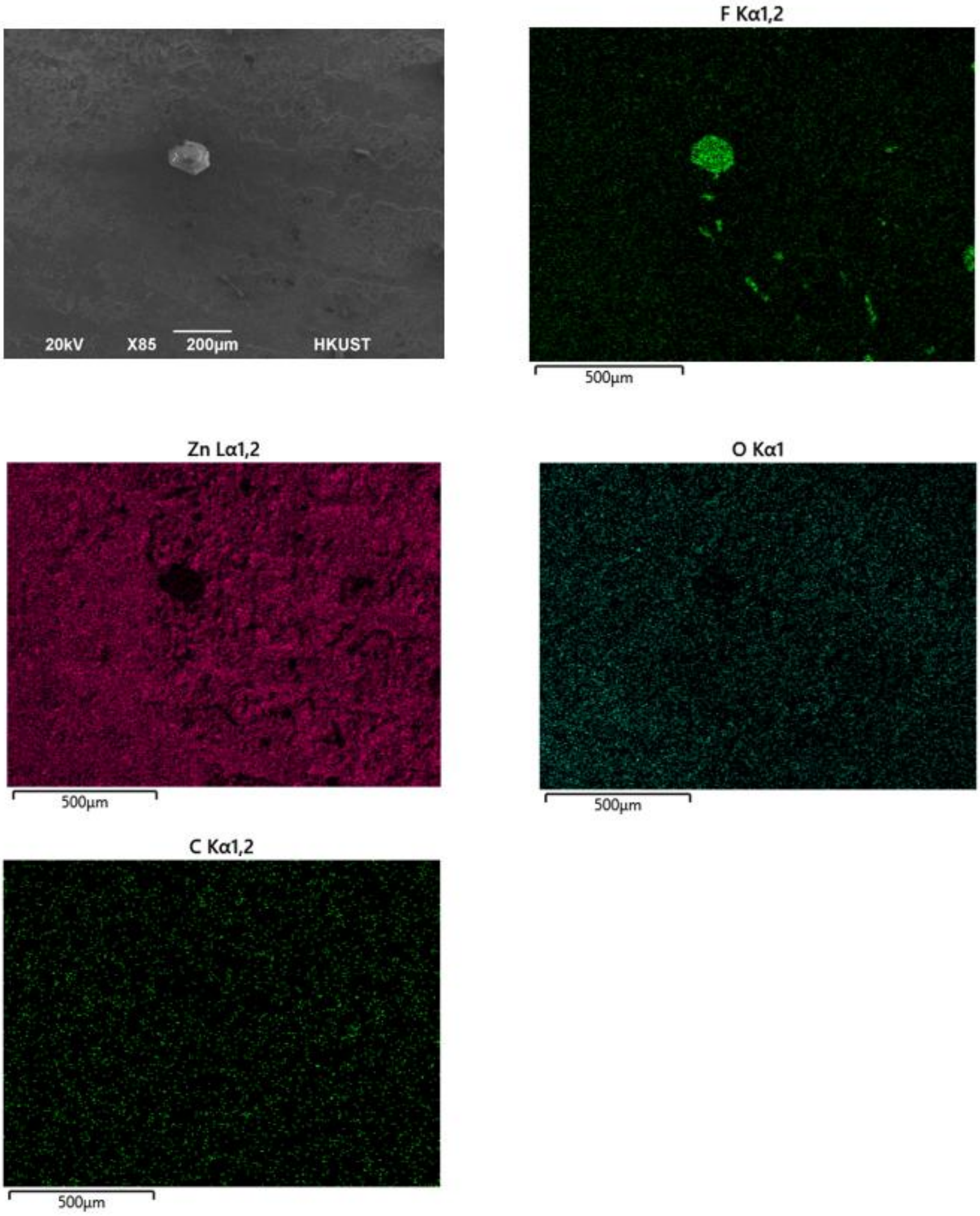

Figure S10. EDX maps of the surface of Zn anodes after cycling in Zn|2M ZBF|Zn symmetric cells.

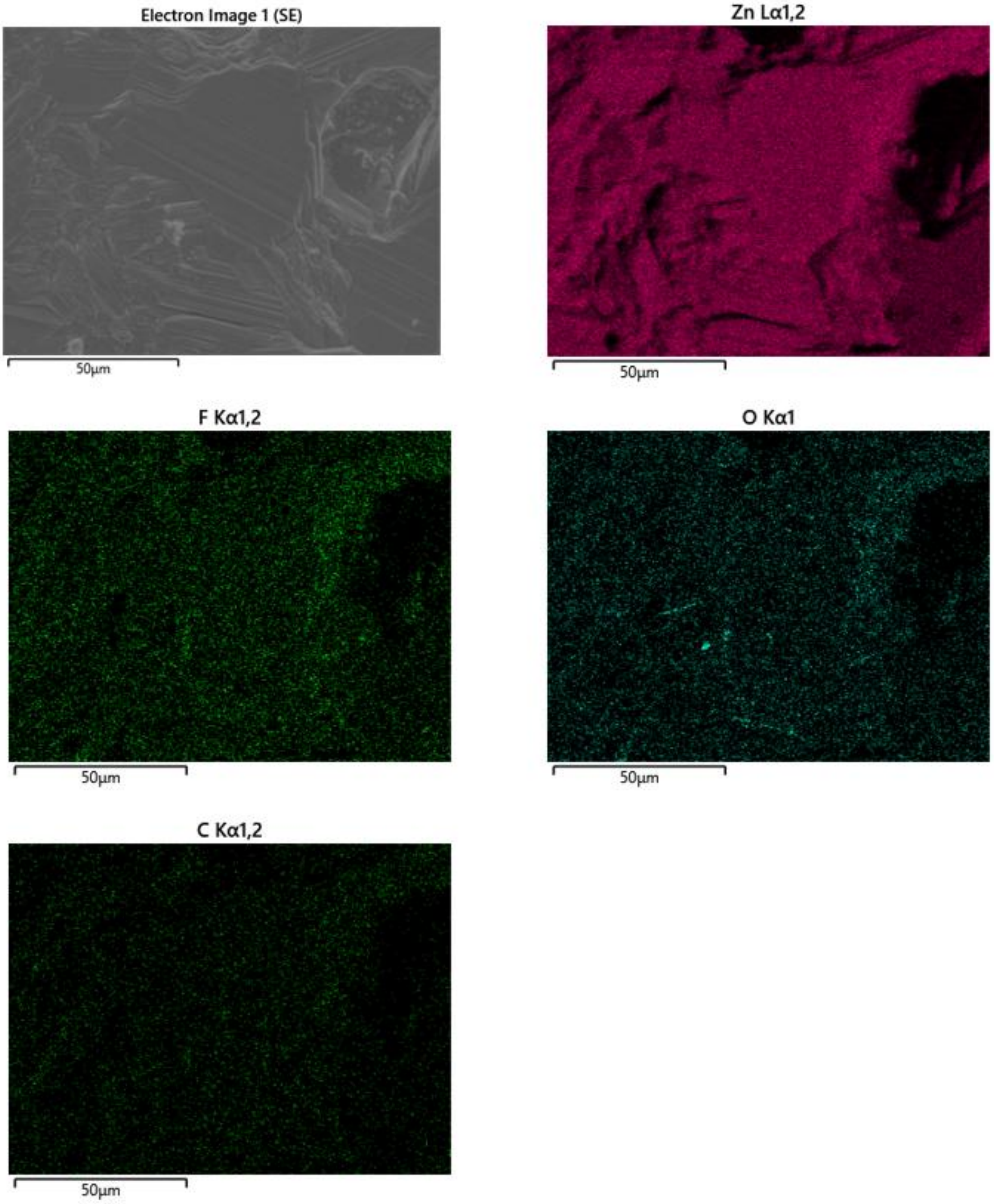

Figure S11. EDX maps of the Zn anode surface after cycling in Zn|ZE-9|Zn symmetric cells for 50 cycles at 0.5 mA/cm^2 and 0.5 mAh/cm^2.

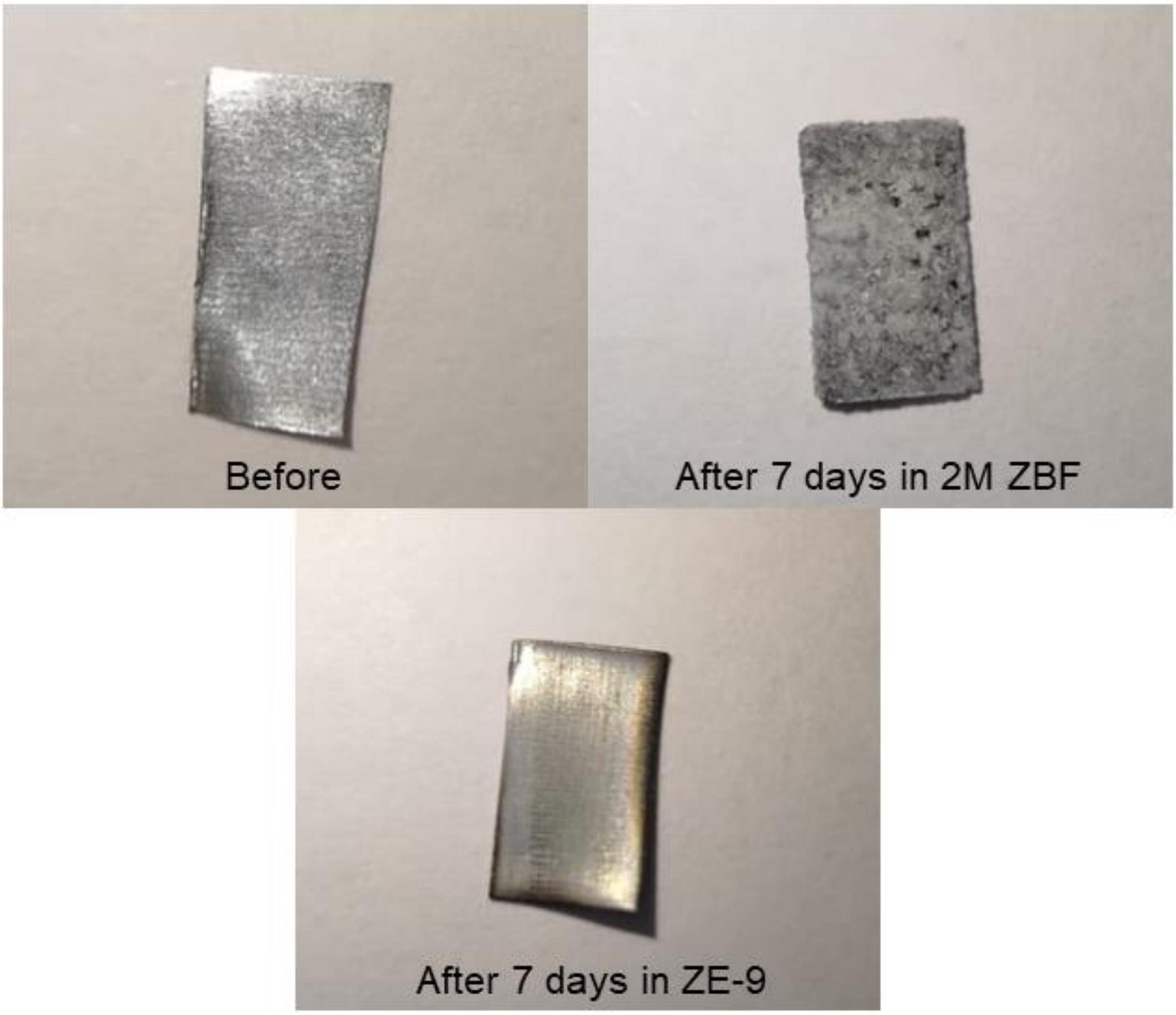

Figure S12. Optical photographs of Zn before and after soaking for 7 days in 2M ZBF and the ZE-9 electrolyte. The 200 μm thick Zn pieces were submerged in electrolyte within a vial. The Zn pieces measure approximately 5 mm x 9 mm.

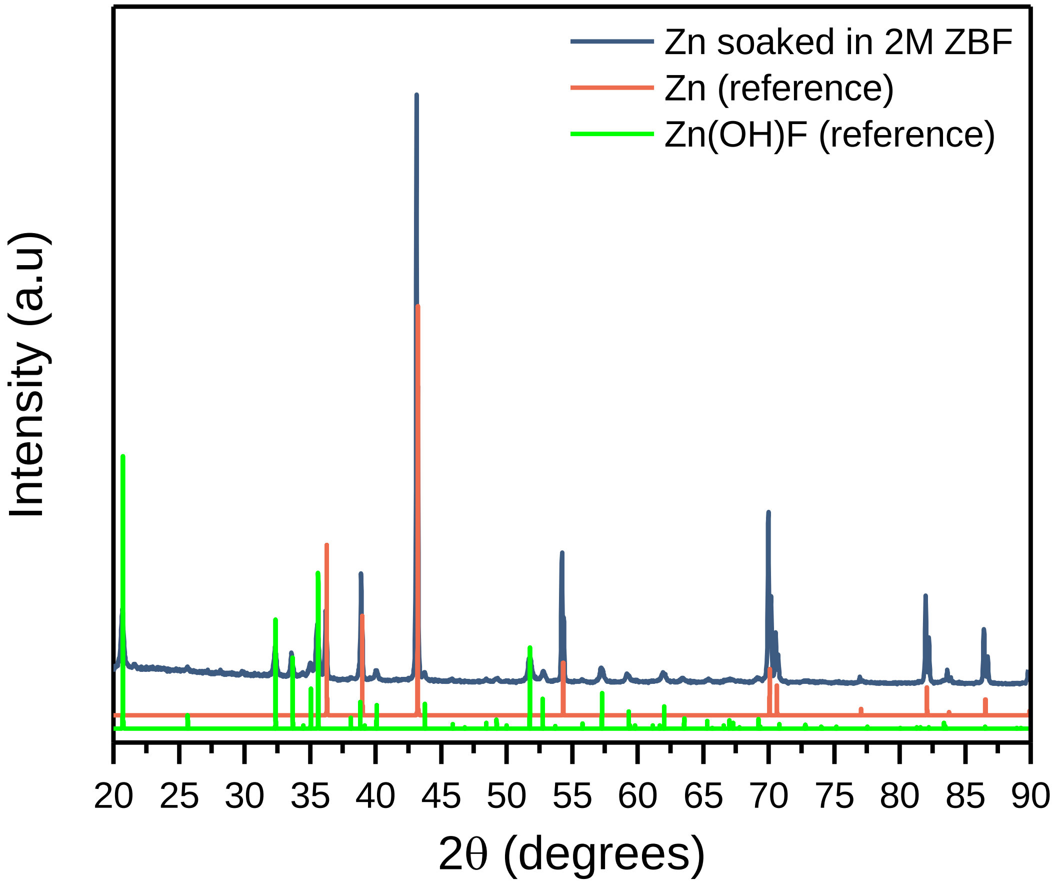

Figure S13: XRD spectra collected from Zn soaked in 2M ZBF for 7 days. Reference spectra for Zn and Zn(OH)F are plotted from .cif files obtained from the Crystallography Open Database. The associated reference numbers are 9008522 for Zn and 9014902 for Zn(OH)F.

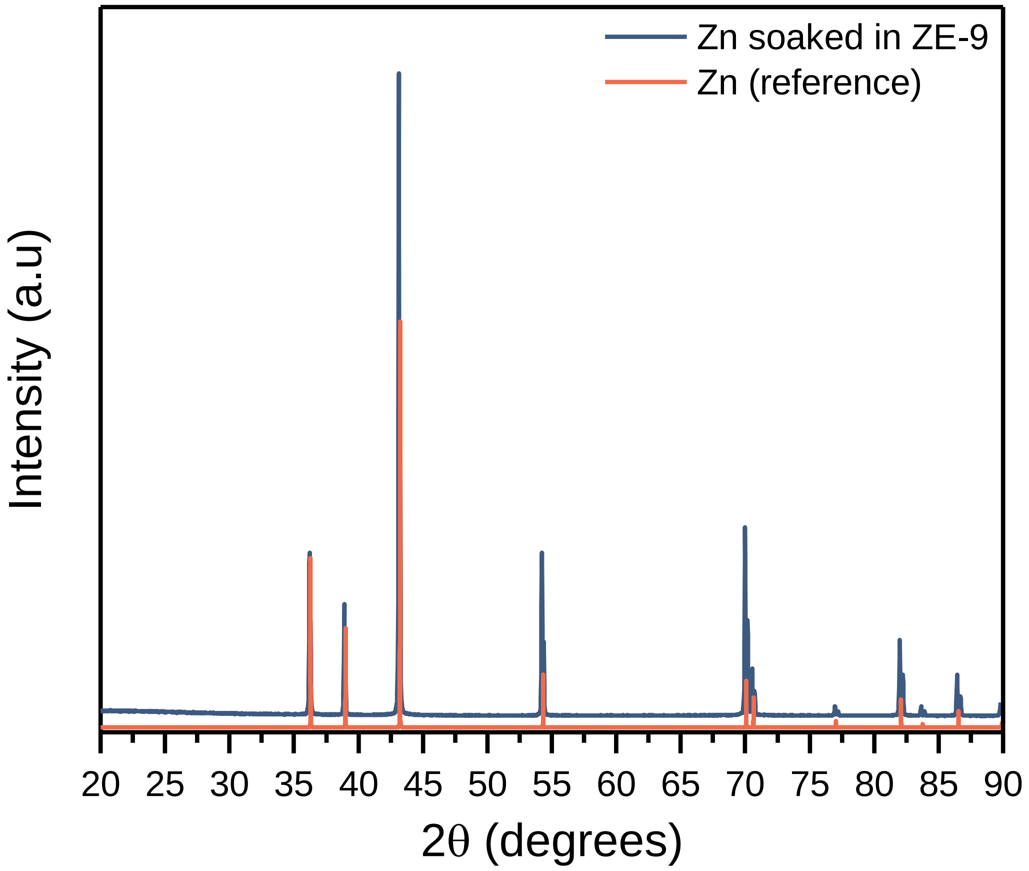

Figure S14: XRD spectra collected from Zn soaked in ZE-9 for 7 days. The reference spectrum for Zn is plotted from a .cif file obtained from the Crystallography Open Database (reference: 9008522).

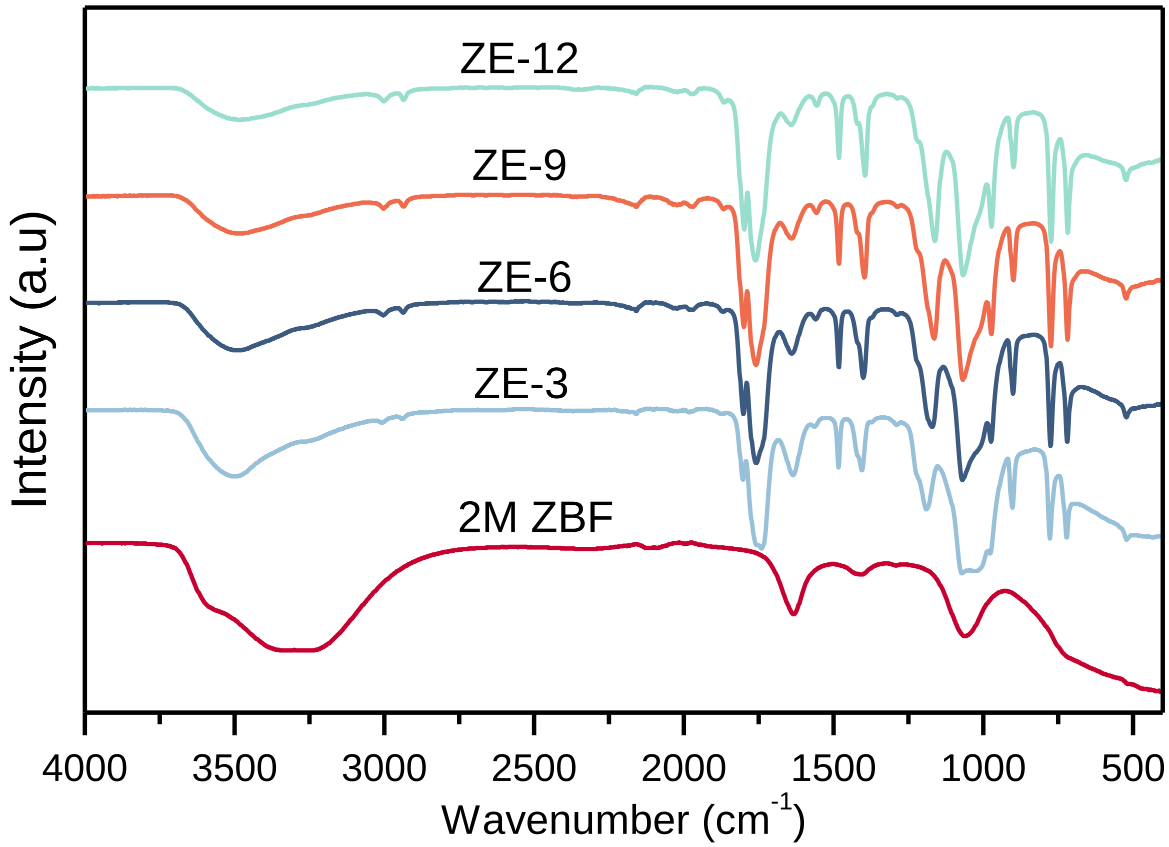

Figure S15. Full FTIR spectra from ZE-x electrolytes, along with the reference 2M ZBF aqueous solution.

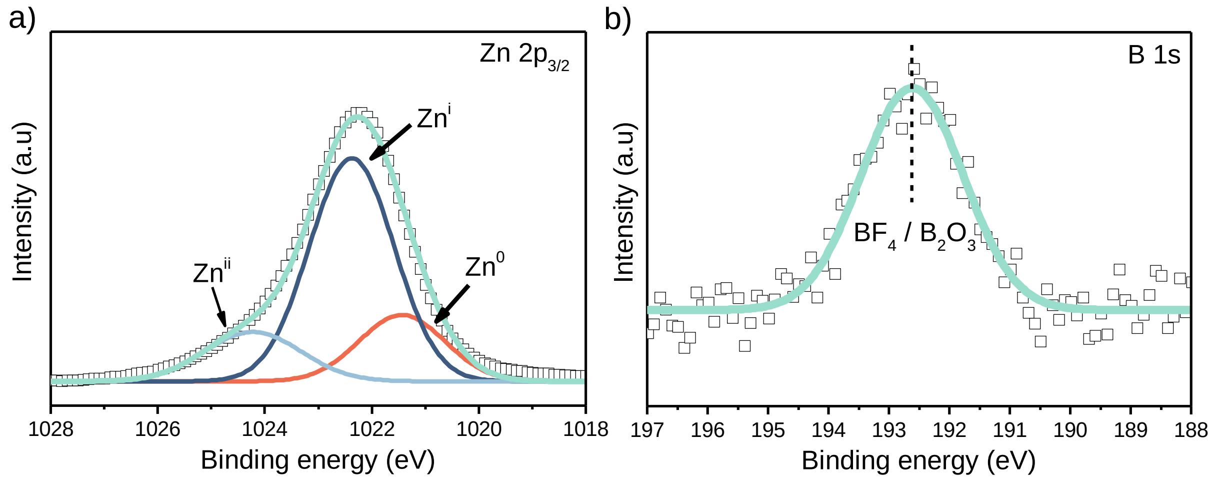

Figure S16. XPS spectra of the a) Zn 2p3/2 and b) B 1s orbitals collected from the surface of Zn anodes cycled for 100 cycles at 3A/g in a Zn|ZE-9|PANI cell. The deconvolved Zn^0, Zn^i and Zn^{ii} subpeaks can be attributed to metallic zinc, interstitial zinc and zinc oxide, and higher order zinc compounds such as ZnF_2 and $Zn(OH)_2$ respectively.

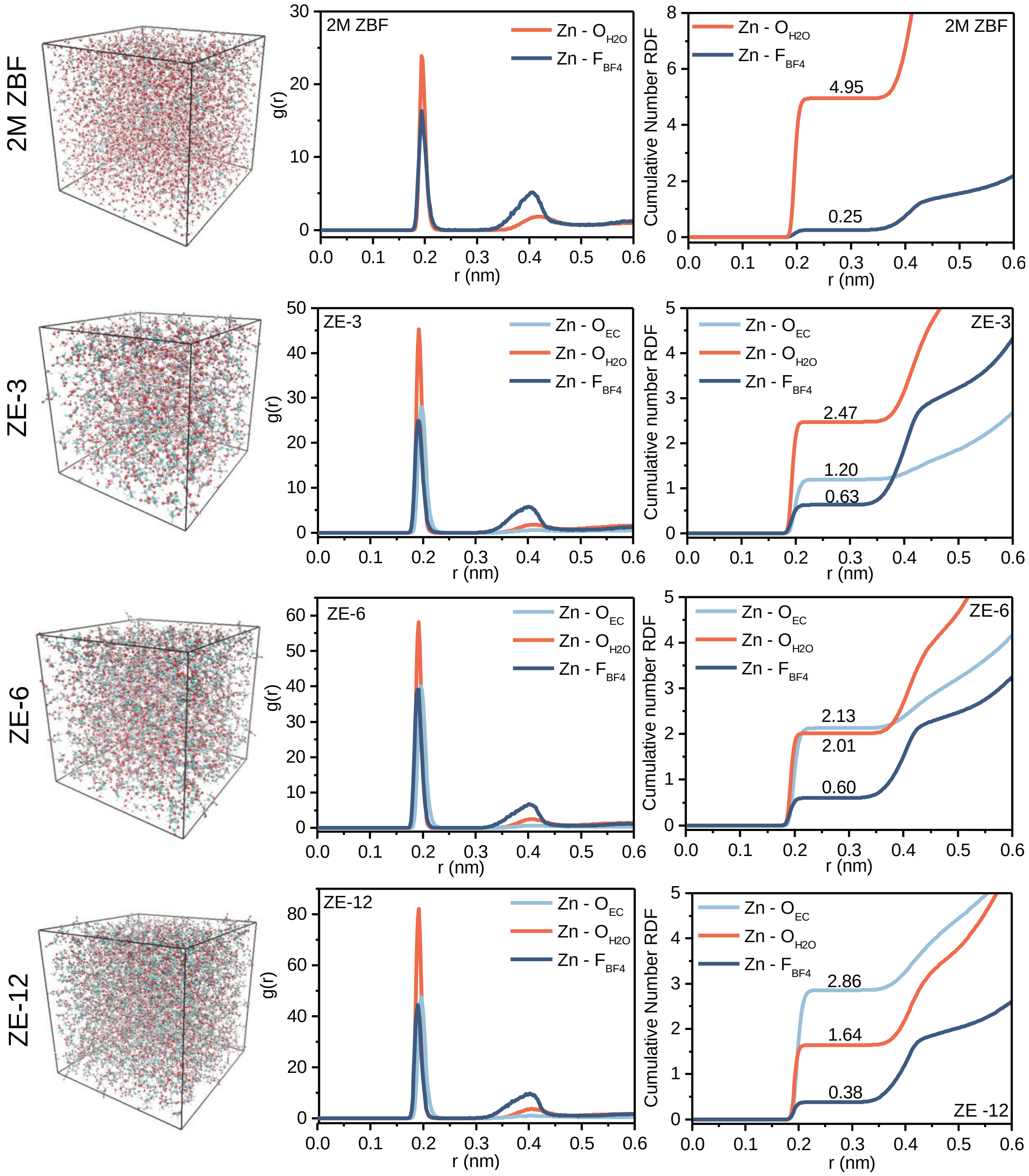

Figure S17. MD snapshots, radial distribution functions and associated coordination number plots for MD simulations of the 2M ZBF, ZE-3, ZE-6 and ZE-12 samples.

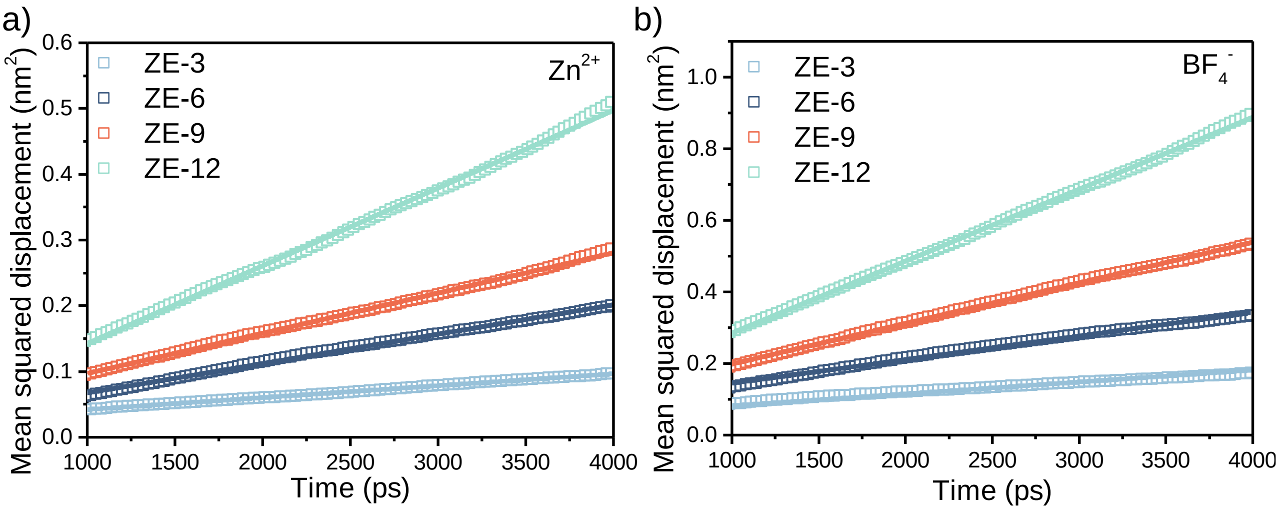

Figure S18. MSD plots for a) Zn^{2+} and b) BF_4^- in the ZE-x electrolyte systems.

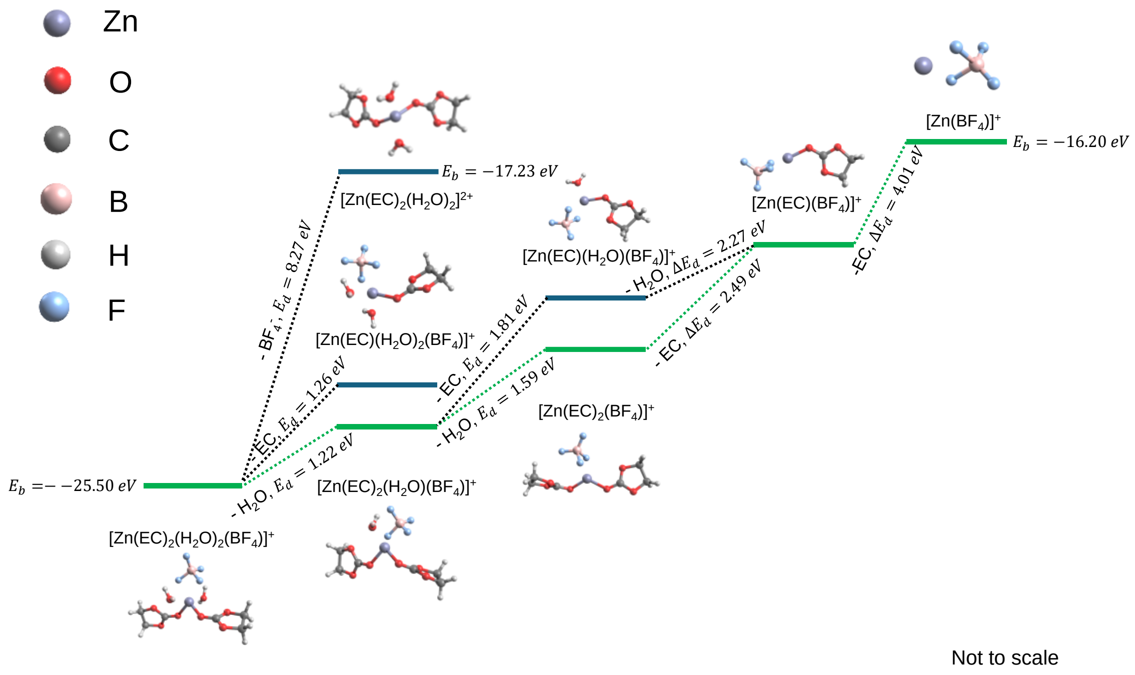

Figure S19. Desolvation energy diagram for the $[Zn(EC)_2(H_2O)_2(BF_4)]^+$ complex identified within the ZE-9 electrolyte system.

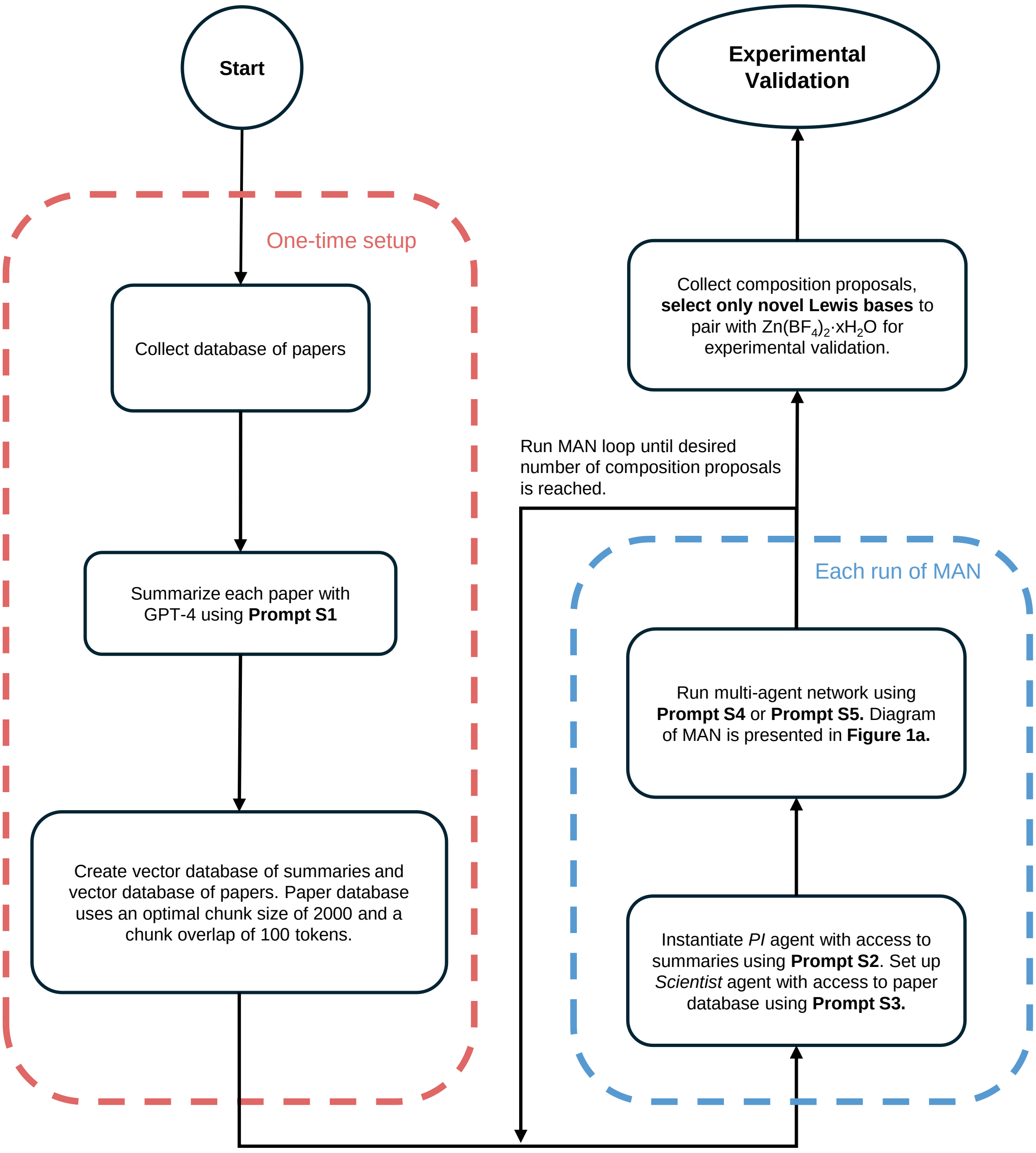

Figure S20. Flowchart depicting the MAN discovery process used in this work, from academic literature collection to composition generation and candidate selection.

Supplementary Tables

Table S1: Complete list of papers used in the input database for the multi-agent network. The papers were selected with a cutoff date of January 2024.

Title	Year	Journal	Ref.
**A ZnCl2 nonaqueous deep-eutectic-solvent electrolyte for zinc-ion hybrid supercapacitors**	2021	Materials Letters	[3]
**Electrodeposition of zinc–tin alloys from deep eutectic solvents based on choline chloride**	2006	Journal of Electroanalytical Chemistry	[4]
**Zinc (II) chloride-based deep eutectic solvents for application as electrolytes: Preparation and characterization**	2015	Journal of Molecular Liquids	[5]
**Moisture-activated deep eutectic electrolyte enabling stable metal Zn anode**	2023	Energy Storage Materials	[6]
**Improving zinc anode reversibility by hydrogen bond in hybrid aqueous electrolyte**	2022	Chemical Engineering Journal	[7]
**Reconstructing anode/electrolyte interface and solvation structure towards high stable zinc anode**	2023	Chemical Engineering Journal	[8]
**Interfacial reconstruction via electronegative sulfonated carbon dots in hybrid electrolyte for ultra-durable zinc battery**	2023	Chemical Engineering Journal	[9]
**Zinc Electrodeposition from deep eutectic solvent containing organic additives**	2017	Journal of Electroanalytical Chemistry	[10]
**Toward stable and highly reversible zinc anodes for aqueous batteries via electrolyte engineering**	2023	Journal of Energy Chemistry	[11]
**Cell-nucleus structured electrolyte for low-temperature aqueous zinc batteries**	2023	Journal of Energy Chemistry	[12]
**“Water-in-deep eutectic solvent” electrolytes enable zinc metal anodes for rechargeable aqueous batteries**	2018	Nano Energy	[13]
**Zinc anode stabilized by an organic-inorganic hybrid solid electrolyte interphase**	2021	Energy Storage Materials	[14]
**Eutectic electrolytes with leveling effects achieving high depth-of-discharge of rechargeable zinc batteries**	2023	Energy Storage Materials	[15]
**Water-in-salt electrolytes towards sustainable and cost-effective alternatives: Example for zinc-ion batteries**	2022	Current Opinion in Electrochemistry	[16]
**A non-flammable hydrous organic electrolyte for sustainable zinc batteries**	2021	Nature Sustainability	[2]
**Eutectic Electrolytes as a Promising Platform for Next-Generation Electrochemical Energy Storage**	2020	Accounts of Chemical Research	[17]
**Electrolytes in Organic Batteries**	2023	Chemical Reviews	[18]
**Reversible Zinc Electrodeposition at −60 °C Using a Deep Eutectic Electrolyte for Low-Temperature Zinc Metal Batteries**	2023	The Journal of Physical Chemistry Letters	[19]
**Ultra-High-Capacity and Dendrite-Free Zinc−Sulfur Conversion Batteries Based on a Low-Cost Deep Eutectic Solvent**	2021	Applied Materials & Interfaces	[20]

**Intercalation of Zinc Monochloride Cations by Deep Eutectic Solvents for High-Performance Rechargeable Non-aqueous Zinc Ion Batteries**	2022	Applied Materials & Interfaces	[21]
**Unraveling the Interphasial Chemistry for Highly Reversible Aqueous Zn Ion Batteries**	2023	Applied Materials & Interfaces	[22]
**Green Environmentally Friendly "$Zn(CH_3SO_3)_2$" Electrolyte for Aqueous Zinc-Ion Batteries**	2023	Applied Materials & Interfaces	[23]
**Chaotropic Anion and Fast-Kinetics Cathode Enabling Low-Temperature Aqueous Zn Batteries**	2021	ACS Energy Letters	[24]
**Eutectic Electrolytes in Advanced Metal-Ion Batteries**	2022	ACS Energy Letters	[25]
**Quantitative Chemistry in Electrolyte Solvation Design for Aqueous Batteries**	2023	ACS Energy Letters	[26]
**Monitoring the Cation Coordination Sphere Using Hydrated Eutectic Electrolyte for Better Cyclic Stability and High Energy Density Zn-Ion Battery**	2023	ACS Sustainable Chemistry & Engineering	[27]
**Tuning the Electrolyte Solvation Structure to Suppress Cathode Dissolution, Water Reactivity, and Zn Dendrite Growth in Zinc-Ion Batteries**	2021	Advanced Functional Materials	[28]
**"Water-in-Deep Eutectic Solvent" Electrolytes for High-Performance Aqueous Zn-Ion Batteries**	2021	Advanced Functional Materials	[29]
**Eutectic Electrolytes with Doubly-Bound Water for High-Stability Zinc Anodes**	2022	Advanced Functional Materials	[30]
**Zincophobic Electrolyte Achieves Highly Reversible Zinc-Ion Batteries**	2023	Advanced Functional Materials	[31]
**Shooting Three Birds with One Stone: The Regulation of Zn2+, H2O, and OH– Kinetics for Stable Zn-Metal Anodes with a Multifunctional Sieve**	2023	Advanced Functional Materials	[32]
**Atomic Insights into Advances and Issues in Low-Temperature Electrolytes**	2023	Advanced Energy Materials	[33]
**Hydrogen-Free and Dendrite-Free All-Solid-State Zn-Ion Batteries**	2020	Advanced Materials	[34]
**Stabilizing Zinc Anodes by Regulating the Electrical Double Layer with Saccharin Anions**	2021	Advanced Materials	[35]
**A New Zinc Salt Chemistry for Aqueous Zinc-Metal Batteries**	2023	Advanced Materials	[36]
**A Zinc–Bromine Battery with Deep Eutectic Electrolytes**	2022	Advanced Science	[37]
**The Effect of Water upon Deep Eutectic Solvent Nanostructure: An Unusual Transition from Ionic Mixture to Aqueous Solution**	2017	Angewandte Chemie	[38]
**Eutectic Electrolyte with Unique Solvation Structure for High Performance Zinc-Ion Batteries**	2022	Angewandte Chemie	[39]
**Comprehensive H_2O Molecules Regulation via Deep Eutectic Solvents for Ultra-Stable Zinc Metal Anode**	2023	Angewandte Chemie	[40]

**High-Capacity Zinc Anode with 96% Utilization Rate Enabled by Solvation Structure Design**	2022	Angewandte Chemie	[41]
**ZnF2-Riched Inorganic/Organic Hybrid SEI: in situ-Chemical Construction and Performance-Improving Mechanism for Aqueous Zinc-ion Batteries**	2022	Angewandte Chemie	[42]
**Low-cost and Non-flammable Eutectic Electrolytes for Advanced Zn-I2 Batteries**	2023	Angewandte Chemie	[43]
**Ultra-stable Zinc Metal Anodes at - 20°C through Eutectic Solvation Sheath in Chlorine-functionalized Eutectic Electrolytes with 1,3-Dioxolane**	2023	Angewandte Chemie	[44]
**Unlocking the Interfacial Adsorption-Intercalation Pseudocapacitive Storage Limit to Enabling All-Climate, High Energy/Power Density and Durable Zn-Ion Batteries**	2023	Angewandte Chemie	[45]
**Triple-function Hydrated Eutectic Electrolyte for Enhanced Aqueous Zinc Batteries**	2023	Angewandte Chemie	[46]
**Deep eutectic solvents: syntheses, properties and applications**	2012	Chem Soc Rev	[47]
**Eutectic-Based Ionic Liquids with Metal-Containing Anions and Cations**	2007	Chemistry Europe	[48]
**Dendrite-Free Engineering toward Efficient Zinc Storage: Recent Progress and Future Perspectives**	2023	Chemistry Europe	[49]
**Microstructural Evolution of Zinc-Ion Species from Aqueous to Hydrated Eutectic Electrolyte for Zn-Ion Batteries**	2023	ChemSusChem	[50]
**Enhanced reversibility and electrochemical window of Zn-ion batteries with an acetonitrile/water-in-salt electrolyte**	2021	Chemical Communications	[51]
**An aqueous hybrid electrolyte for low-temperature zinc-based energy storage devices**	2020	Energy & Environmental Science	[52]
**Regulation methods for the Zn/electrolyte interphase and the effectiveness evaluation in aqueous Zn-ion batteries**	2021	Energy & Environmental Science	[53]
**Non-flammable, dilute, and hydrous organic electrolytes for reversible Zn batteries**	2022	Chemical Science	[54]
**A flexible and highly ion conductive polyzwitterionic eutectogel for quasi-solid state zinc ion batteries with efficient suppression of dendrite growth**	2022	Journal of Materials Chemistry A	[55]
**A hydrated deep eutectic electrolyte with finely-tuned solvation chemistry for high-performance zinc-ion batteries**	2023	Energy & Environmental Science	[56]
**Interphases in aqueous rechargeable zinc metal batteries**	2023	Journal of Materials Chemistry A	[57]
**Zn-Based Deep Eutectic Solvent as the Stabilizing Electrolyte for Zn Metal Anode in Rechargeable Aqueous Batteries**	2022	Frontiers in Chemistry	[58]
**Rechargeable Zinc-Ion Battery Based on Choline Chloride-Urea Deep Eutectic Solvent**	2019	Journal of The Electrochemical Society	[59]

**Hydrated Deep Eutectic Electrolytes for High-Performance Zn-Ion Batteries Capable of Low-Temperature Operation**	2022	Advanced Functional Materials	[60]
**Zinc Electrode Cycling in Deep Eutectic Solvent Electrolytes: An Electrochemical Study**	2023	Molecules	[61]
**Eutectic Electrolytes Chemistry for Rechargeable Zn Batteries**	2022	Small	[62]
**Synergistic Effect of Cation and Anion for Low-Temperature Aqueous Zinc-Ion Battery**	2021	Nano-Micro Letters	[63]
**Zinc anode-compatible in-situ solid electrolyte interphase via cation solvation modulation**	2019	Nature Communications	[64]
**Tailoring water structure with high tetrahedral-entropy for antifreezing electrolytes and energy storage at −80 °C**	2022	Nature Communications	[65]
**Sulfolane-containing aqueous electrolyte solutions for producing efficient amperehour-level zinc metal battery pouch cells**	2023	Nature Communications	[66]
**Enabling selective zinc-ion intercalation by a eutectic electrolyte for practical anodeless zinc batteries**	2023	Nature Communications	[67]
**Hydrotropic solubilization of zinc acetates for sustainable aqueous battery electrolytes**	2023	Nature Sustainability	[68]
**Regulating the solvation sheath of zinc ions by supramolecular coordination chemistry toward ultrastable zinc anodes**	2023	SmartMat	[69]
**Mechanistic Studies of Zinc Electrodeposition from Deep Eutectic Electrolytes**	2014	Journal of The Electrochemical Society	[70]
**Zinc Electrodeposition from a Deep Eutectic System Containing Choline Chloride and Ethylene Glycol**	2010	Journal of The Electrochemical Society	[71]
**Aqueous aluminum-zinc hybrid ion batteries with urea-based hydrated eutectic electrolytes**	2024	Nano Energy	[72]
**Ternary eutectic electrolytes attune the electrode/electrolyte interphase layer toward long-life zinc ion batteries**	2024	Energy Storage Materials	[73]
**Tetrabutylammonium bromide incorporated hydrated deep eutectic solvents: Simultaneously addressing anode stability and cathode efficiency in zinc-bromine batteries**	2024	Energy Storage Materials	[74]
**Extending temperature windows of practical zinc batteries by water-locking hydrated eutectic solvents**	2024	Energy Storage Materials	[75]
**Rotational dynamics, ionic conductivity, and glass formation in a ZnCl2-based deep eutectic solvent**	2024	The Journal of Chemical Physics	[76]
**Acetamide-Caprolactam Deep Eutectic Solvent-Based Electrolyte for Stable Zn-Metal Batteries**	2023	Advanced Materials	[77]
**Competitive Coordination Structure Regulation in Deep Eutectic Electrolyte for Stable Zinc Batteries**	2024	Angewandte Chemie	[78]
**Solvation structure regulation of deep eutectic solvents: stabilization of the zinc anode in rechargeable zinc–air batteries**	2024	Journal of Materials Chemistry A	[79]

**Effect of Water on Zn Electrodeposition from a Deep Eutectic Solvent**	2024	Journal of The Electrochemical Society	[80]

Table S2: Generated electrolyte compositions from the multi-agent network

Iteration	Prompt	Component 1	Component 2	Component 3	Component 4
1	Prompt 4	$Zn(TFSI)_2$	Ethylene Glycol	Levulinic acid	
2	Prompt 4	$Zn(OTF)_2$	Choline acetate	Erythritol	Water
3	Prompt 5	$Zn(BF_4)_2 \cdot xH_2O$	Ethylene Carbonate	LiTFSI	
4	Prompt 5	$Zn(BF_4)_2 \cdot xH_2O$	Glycerol	Water	
5	Prompt 5	$Zn(BF_4)_2 \cdot xH_2O$	Lactic acid	Betaine	

Table S3: Full list of tested compositions using Lewis bases suggested by the MAN. A tick represents a successfully formed DEE in a visually homogeneous liquid state at room temperature. In contrast, a cross denotes that the electrolyte contained either visible solid particles or was fully solid at that molar ratio at room temperature.

	Molar Ratio (ZBF:X)						
	**3:2**	**1:1**	**2:3**	**1:3**	**1:6**	**1:9**	**1:12**
ZBF:EC	✗	✗	✗	✓	✓	✓	✓
ZBF:ERY	✓	✓	✓	✗	✗	✗	✗
ZBF:GLY	✗	✗	✗	✓	✓	✓	✓
ZBF:LEV	✗	✗	✗	✓	✓	✓	✓
ZBF:LAC	✗	✗	✗	✓	✓	✓	✓

Table S4: Performance comparison between the conductivity and 5 A/g rate performance of the electrolyte in this work and other deep eutectic electrolyte and aqueous electrolyte systems.

Electrolyte composition	Molar Ratio	Cathode	Conductivity (mS/cm)	Rate performance	Reference
**ZBF + EC**	**1:9 ZBF:EC**	**polyaniline**	**10.6**	**81 mAh/g @ 5 A/g**	**This work**
ZBF + ethylene glycol	4M ZBF in ethylene glycol (approx. 1:5.5 ZBF:ethylene glycol)	V_2O_5	4.5	Not tested at 5 A/g	Han et al.[2]
ZBF + acetamide	1:8 ZBF:acetamide	polyaniline	4.13	61.4 mAh/g @ 5 A/g	Wang et al.[81]
ZBF + sulfolane	1:2 ZBF:sulfolane	$NH_4V_4O_{10}$	1.8	68 mAh/g @ 5 A/g	Li et al.[82]
$Zn(ClO_4)_2.6H_2O$ + sulfolane	1:6 $Zn(ClO_4)_2.6H_2O$:sulfolane	polyaniline	2.0	38 mAh/g @ 5 A/g	Lin et al.[60]
Methyl urea + $Na(ClO)_4$ + $ZnCl_2$ + water + acetonitrile	4:1:0.05:2 Methyl urea: $Na(ClO)_4$:$ZnCl_2$:water + 40% acetonitrile by volume	Polyaniline decorated graphene nanofibers	16.08	Not tested at 5 A/g. 43 mAh/g @ 2 A/g	Kumar et al.[83]
$Zn(OTF)_2$ + LiTFSI + N-methyl acetamide	2:1:9 $Zn(OTF)_2$: LiTFSI: N-methyl acetamide	polyaniline	-	50 mAh/g @ 5 A/g	Li et al.[84]
$Zn(CF_3SO_3)_2$ + water	1M $Zn(CF_3SO_3)_2$ in water	polyaniline	-	95 mAh/g @ 5 A/g	Wan et al.[85]
$ZnCl_2$ + ethylene glycol + polyvinyl alcohol (PVA) + water	11 wt% PVA + 10 wt% water in 1:4 $ZnCl_2$:ethylene glycol.	Polyaniline carbon cloth	4.5	72 mAh/g @ 5 A/g	Peng et al.[86]

Table S5: Performance comparison between this work and other deep eutectic electrolyte systems. The selected comparison electrolytes are all binary hydrated deep eutectic electrolytes, where water is provided only by the hydrated zinc salt.

**Electrolyte Composition**	**Molar Ratio**	**Coulombic Efficiency**	**Cycling Stability in Zn||Zn cells**	**Full Cell Cycling**	**Reference**
ZBF + EC	1:9 ZBF:EC	98.89% in Zn||SS cells at 0.5 mA/cm^2, 0.5 mAh/cm^2	700h @ 0.5 mA/cm^2 and 0.25 mAh/cm^2	600 cycles at 1A/g with 71% capacity retention in Zn||PANI cells. n/p ratio 7.1. Mass loading 8.6 mg/cm2	**This work**
ZBF + ethylene glycol	4M ZBF in ethylene glycol	99.4 % in Zn||Cu cells at 1 mA/cm^2 and 0.5 mAh/cm^2	4000 h @ 0.5 mA/cm^2 and 0.25 mAh/cm^2	800 cycles in Zn||V2O5 cell at 1 A/g with ~ 60% capacity retention. n/p ratio 17-25. Mass loading 2-3 mg/cm^2	Han et al.[2]
ZBF + acetamide	1:8 ZBF:acetamide	99.53% in Zn||Cu cells at 2 mA/cm^2 and 0.5 mAh/cm^2	4400h @ 0.5 mA/cm^2 and 0.25 mAh/cm^2 in	4000 cycles in Zn||PANI cells at 1A/g with 73.2% capacity retention. Mass loading 1.5-2.0 mg/cm^2	Wang et al.[81]
ZBF + sulfolane	1:2 ZBF:sulfolane	99.5% in Zn||Cu cells At 1 mA/cm^2 and 1 mAh/cm^2	1650 h @ 2 mA/cm^2 and 1 mAh/cm^2	1000 cycles in Zn||$NH_4V_4O_{10}$ cells with 83.4% capacity retention at 1 A/g. Mass loading 1.4-1.6 mg/cm^2	Li et al.[82]
Zinc perchlorate hexahydrate + sulfolane	1:6 $Zn(ClO_4)_2.6H_2O$: sulfolane	98% in Zn||SS cells at 0.5 mA/cm^2 and 0.5 mAh/cm^2	800 h @ 0.5 mA/cm^2 and 0.5 mAh/cm^2	2500 cycles at 3 A/g in Zn||PANI cells with ~ 75% capacity retention. Mass loading 1.0-1.5 mg/cm^2	Lin et al.[60]
Zinc perchlorate hexahydrate + succinonitrile	1:8 $Zn(ClO_4)_2.6H_2O$: succinonitrile	98.4% in Zn||SS cells at 0.5 mA/cm^2 and 0.5 mAh/cm^2	800h @ 0.2 mA/cm^2 and 0.5 mAh/cm^2	3500 cycles at 0.15 C in Zn|| Poly(2,3-dithiin-1,4-benzoquinone cell with 87% capacity retention	Yang et al.[87]

References

[1] "Ragas", https://github.com/explodinggradients/ragas, accessed 11, **2024.**
[2] D. Han, C. Cui, K. Zhang, Z. Wang, J. Gao, Y. Guo, Z. Zhang, S. Wu, L. Yin, Z. Weng, F. Kang, Q.-H. Yang, *Nat. Sustain.* **2022**, *5*, 205.
[3] H. Tian, R. Cheng, L. Zhang, Q. Fang, P. Ma, Y. Lv, F. Wei, *Mater. Lett.* **2021**, *301*, 130237.
[4] A. P. Abbott, G. Capper, K. J. McKenzie, K. S. Ryder, *J. Electroanal. Chem.* **2007**, *599*, 288.
[5] F. S. Ghareh Bagh, K. Shahbaz, F. S. Mjalli, M. A. Hashim, I. M. AlNashef, *J. Mol. Liq.* **2015**, *204*, 76.
[6] Y. Xie, J. Huang, T. Kong, X. Zhou, K. Wu, X. Liu, J. Yi, L. Xing, Y. Xia, *Energy Storage Mater.* **2023**, *56*, 218.
[7] H. Du, K. Wang, T. Sun, J. Shi, X. Zhou, W. Cai, Z. Tao, *Chem. Eng. J.* **2022**, *427*, 131705.
[8] T. Wei, X. Zhang, Y. Ren, Y. Wang, Z. Li, H. Zhang, L. Hu, *Chem. Eng. J.* **2023**, *457*, 141272.
[9] H. Zhang, Z. Luo, W. Deng, J. Hu, G. Zou, H. Hou, X. Ji, *Chem. Eng. J.* **2023**, *461*, 142105.
[10] N. M. Pereira, C. M. Pereira, J. P. Araújo, A. Fernando Silva, *J. Electroanal. Chem.* **2017**, *801*, 545.
[11] A. Li, J. Li, Y. He, M. Wu, *J. Energy Chem.* **2023**, *83*, 209.
[12] Y. Dong, N. Zhang, Z. Wang, J. Li, Y. Ni, H. Hu, F. Cheng, *J. Energy Chem.* **2023**, *83*, 324.
[13] J. Zhao, J. Zhang, W. Yang, B. Chen, Z. Zhao, H. Qiu, S. Dong, X. Zhou, G. Cui, L. Chen, *Nano Energy* **2019**, *57*, 625.
[14] S. Di, X. Nie, G. Ma, W. Yuan, Y. Wang, Y. Liu, S. Shen, N. Zhang, *Energy Storage Mater.* **2021**, *43*, 375.
[15] J. Wang, H. Qiu, Q. Zhang, X. Ge, J. Zhao, J. Wang, Y. Ma, C. Fan, X. Wang, Z. Chen, G. Li, G. Cui, *Energy Storage Mater.* **2023**, *58*, 9.
[16] T. F. Burton, R. Jommongkol, Y. Zhu, S. Deebansok, K. Chitbankluai, J. Deng, O. Fontaine, *Curr. Opin. Electrochem.* **2022**, *35*, 101070.
[17] C. Zhang, L. Zhang, G. Yu, *Acc. Chem. Res.* **2020**, *53*, 1648.
[18] M. Li, R. P. Hicks, Z. Chen, C. Luo, J. Guo, C. Wang, Y. Xu, *Chem. Rev.* **2023**, *123*, 1712.
[19] B. E. Hawkins, T. Schoetz, L. W. Gordon, S. Kt, J. Wang, R. J. Messinger, *J. Phys. Chem. Lett.* **2023**, *14*, 2378.
[20] M. Cui, J. Fei, F. Mo, H. Lei, Y. Huang, *ACS Appl. Mater. Interfaces* **2021**, *13*, 54981.
[21] S.-C. Wu, M.-C. Tsa, H.-J. Liao, T.-Y. Su, S.-Y. Tang, C.-W. Chen, H.-A. Lo, T.-Y. Yang, K. Wang, Y. Ai, Y.-Z. Chen, L. Lee, J.-F. Lee, C.-J. Lin, B. J. Hwang, Y.-L. Chueh, *ACS Appl. Mater. Interfaces* **2022**, *14*, 7814.
[22] X. Zhao, N. Dong, M. Yan, H. Pan, *ACS Appl. Mater. Interfaces* **2023**, *15*, 4053.
[23] C. Sun, R. Miao, J. Li, Y. Sun, Y. Chen, J. Pan, Y. Tang, P. Wan, *ACS Appl. Mater. Interfaces* **2023**, *15*, 20089.
[24] Q. Zhang, K. Xia, Y. Ma, Y. Lu, L. Li, J. Liang, S. Chou, J. Chen, *ACS Energy Lett.* **2021**, *6*, 2704.
[25] L. Geng, X. Wang, K. Han, P. Hu, L. Zhou, Y. Zhao, W. Luo, L. Mai, *ACS Energy Lett.* **2022**, *7*, 247.

[26] L. Li, H. Cheng, J. Zhang, Y. Guo, C. Sun, M. Zhou, Q. Li, Z. Ma, J. Ming, *ACS Energy Lett.* **2023**, *8*, 1076.
[27] P. Samanta, S. Ghosh, A. Kundu, H. Kolya, C.-W. Kang, P. Samanta, N. C. Murmu, T. Kuila, *ACS Sustain. Chem. Eng.* **2023**, *11*, 5495.
[28] S. Liu, J. Mao, W. K. Pang, J. Vongsvivut, X. Zeng, L. Thomsen, Y. Wang, J. Liu, D. Li, Z. Guo, *Adv. Funct. Mater.* **2021**, *31*, 2104281.
[29] J. Shi, T. Sun, J. Bao, S. Zheng, H. Du, L. Li, X. Yuan, T. Ma, Z. Tao, *Adv. Funct. Mater.* **2021**, *31*, 2102035.
[30] D. Han, T. Sun, R. Zhang, W. Zhang, T. Ma, H. Du, Q. Wang, D. He, S. Zheng, Z. Tao, *Adv. Funct. Mater.* **2022**, *32*, 2209065.
[31] N. Wang, X. Chen, H. Wan, B. Zhang, K. Guan, J. Yao, J. Ji, J. Li, Y. Gan, L. Lv, L. Tao, G. Ma, H. Wang, J. Zhang, H. Wang, *Adv. Funct. Mater.* **2023**, *33*, 2300795.
[32] H. Zhang, Y. Wu, J. Yu, T. Jiang, M. Wu, *Adv. Funct. Mater.* **2024**, *34*, 2301912.
[33] R. Hou, S. Guo, H. Zhou, *Adv. Energy Mater.* **2023**, *13*, 2300053.
[34] L. Ma, S. Chen, N. Li, Z. Liu, Z. Tang, J. A. Zapien, S. Chen, J. Fan, C. Zhi, *Adv. Mater.* **2020**, *32*, 1908121.
[35] C. Huang, X. Zhao, S. Liu, Y. Hao, Q. Tang, A. Hu, Z. Liu, X. Chen, *Adv. Mater.* **2021**, *33*, 2100445.
[36] H. Du, Y. Dong, Q. Li, R. Zhao, X. Qi, W. Kan, L. Suo, L. Qie, J. Li, Y. Huang, *Adv. Mater.* **2023**, *35*, 2210055.
[37] J. Heo, K. Shin, H. Kim, *Adv. Sci.* **2022**, *9*, 2204908.
[38] O. S. Hammond, D. T. Bowron, K. J. Edler, *Angew. Chem. Int. Ed.* **2017**, *56*, 9782.
[39] L. Geng, J. Meng, X. Wang, C. Han, K. Han, Z. Xiao, M. Huang, P. Xu, L. Zhang, L. Zhou, L. Mai, *Angew. Chem. Int. Ed.* **2022**, *61*, e202206717.
[40] M. Li, X. Wang, J. Hu, J. Zhu, C. Niu, H. Zhang, C. Li, B. Wu, C. Han, L. Mai, *Angew. Chem. Int. Ed.* **2023**, *62*, e202215552.
[41] M. Wang, J. Ma, Y. Meng, J. Sun, Y. Yuan, M. Chuai, N. Chen, Y. Xu, X. Zheng, Z. Li, W. Chen, *Angew. Chem. Int. Ed.* **2022**, *62*, e202214966.
[42] D. Xie, Y. Sang, D. Wang, W. Diao, F. Tao, C. Liu, J. Wang, H. Sun, J. Zhang, X. Wu, *Angew. Chem. Int. Ed.* **2023**, *62*, e202216934.
[43] J. Hao, L. Yuan, Y. Zhu, X. Bai, C. Ye, Y. Jiao, S. Qiao, *Angew. Chem. Int. Ed.* **2023**, *62*, e202310284.
[44] X. Lu, Z. Liu, A. Amardeep, Z. Wu, L. Tao, K. Qu, H. Sun, Y. Liu, J. Liu, *Angew. Chem. Int. Ed.* **2023**, *62*, e202307475.
[45] M. Yang, Y. Wang, D. Ma, J. Zhu, H. Mi, Z. Zhang, B. Wu, L. Zeng, M. Chen, J. Chen, P. Zhang, *Angew. Chem. Int. Ed.* **2023**, *62*, e202304400.
[46] Y. Zhong, X. Xie, Z. Zeng, B. Lu, G. Chen, J. Zhou, *Angew. Chem. Int. Ed.* **2023**, *62*, e202310577.
[47] Q. Zhang, K. D. O. Vigier, S. Royer, F. Jérôme, *Chem. Soc. Rev.* **2012**, *41*, 7108.
[48] A. P. Abbott, J. C. Barron, K. S. Ryder, D. Wilson, *Chem. – Eur. J.* **2007**, *13*, 6495.
[49] L. Miao, J. Zhang, Y. Lv, L. Gan, M. Liu, *Chem. Eur. J.* **2023**, *29*, DOI 10.1002/chem.202203973.
[50] L. Su, F. Lu, Y. Li, X. Li, L. Chen, Y. Gao, L. Zheng, X. Gao, *ChemSusChem* **2023**, *16*, e202300285.
[51] X. Song, H. He, M. H. Aboonasr Shiraz, H. Zhu, A. Khosrozadeh, J. Liu, *Chem. Commun.* **2021**, *57*, 1246.
[52] N. Chang, T. Li, R. Li, S. Wang, Y. Yin, H. Zhang, X. Li, *Energy Environ. Sci.* **2020**, *13*, 3527.
[53] L. Yuan, J. Hao, C.-C. Kao, C. Wu, H.-K. Liu, S.-X. Dou, S.-Z. Qiao, *Energy Environ. Sci.* **2021**, *14*, 5669.

[54] G. Ma, L. Miao, W. Yuan, K. Qiu, M. Liu, X. Nie, Y. Dong, N. Zhang, F. Cheng, *Chem. Sci.* **2022**, *13*, 11320.
[55] Y. Wu, Y. Deng, K. Zhang, Y. Wang, L. Wang, L. Yan, *J. Mater. Chem. A* **2022**, *10*, 17721.
[56] R. Chen, C. Zhang, J. Li, Z. Du, F. Guo, W. Zhang, Y. Dai, W. Zong, X. Gao, J. Zhu, Y. Zhao, X. Wang, G. He, *Energy Environ. Sci.* **2023**, *16*, 2540.
[57] R. Jayakumar, D. M. Harrison, J. Xu, A. V. Suresh Babu, C. Luo, L. Ma, *J. Mater. Chem. A* **2023**, *11*, 8470.
[58] G. M. Thorat, V.-C. Ho, J. Mun, *Front. Chem.* **2022**, *9*, 825807.
[59] W. Kao-ian, R. Pornprasertsuk, P. Thamyongkit, T. Maiyalagan, S. Kheawhom, *J. Electrochem. Soc.* **2019**, *166*, A1063.
[60] X. Lin, G. Zhou, M. J. Robson, J. Yu, S. C. Kwok, F. Ciucci, *Adv. Funct. Mater.* **2021**, 2109322.
[61] E. Emanuele, A. Li Bassi, A. Macrelli, C. Mele, J. Strada, B. Bozzini, *Molecules* **2023**, *28*, 957.
[62] X. Lu, E. J. Hansen, G. He, J. Liu, *Small* **2022**, *18*, 2200550.
[63] T. Sun, S. Zheng, H. Du, Z. Tao, *Nano-Micro Lett.* **2021**, *13*, 204.
[64] H. Qiu, X. Du, J. Zhao, Y. Wang, J. Ju, Z. Chen, Z. Hu, D. Yan, X. Zhou, G. Cui, *Nat. Commun.* **2019**, *10*, 5374.
[65] M. Qiu, P. Sun, K. Han, Z. Pang, J. Du, J. Li, J. Chen, Z. L. Wang, W. Mai, *Nat. Commun.* **2023**, *14*, 601.
[66] Y. Wang, T. Wang, S. Bu, J. Zhu, Y. Wang, R. Zhang, H. Hong, W. Zhang, J. Fan, C. Zhi, *Nat. Commun.* **2023**, *14*, 1828.
[67] C. Li, R. Kingsbury, A. S. Thind, A. Shyamsunder, T. T. Fister, R. F. Klie, K. A. Persson, L. F. Nazar, *Nat. Commun.* **2023**, *14*, 3067.
[68] D. Dong, T. Wang, Y. Sun, J. Fan, Y.-C. Lu, *Nat. Sustain.* **2023**, *6*, 1474.
[69] G. Zhang, Y. Chen, L. Fu, L. Zheng, K. Fan, C. Zhang, J. Zou, H. Dai, L. Guan, Y. Cao, M. Mao, J. Ma, C. Wang, *SmartMat* **2024**, *5*, e1216.
[70] L. Vieira, A. H. Whitehead, B. Gollas, *J. Electrochem. Soc.* **2013**, *161*, D7.
[71] A. H. Whitehead, M. Pölzler, B. Gollas, *J. Electrochem. Soc.* **2010**, *157*, D328.
[72] C. Lu, Z. Wang, Y. Zhang, G. Tang, Y. Wang, X. Guo, J. Li, L. Wei, *Nano Energy* **2024**, *120*, 109158.
[73] W. Li, W. Kong, W. Liu, S. Xu, H. Zhu, S. Liu, W. Yu, Z. Wen, *Energy Storage Mater.* **2024**, *65*, 103103.
[74] Y. Lim, G. Lee, J. H. Kim, J. K. Seo, S. J. Yoo, *Energy Storage Mater.* **2024**, *68*, 103331.
[75] N. Wang, M. Sun, X. Ren, L. Hu, Z. Li, X. Yao, C. Jia, *Energy Storage Mater.* **2024**, *68*, 103333.
[76] A. Schulz, P. Lunkenheimer, A. Loidl, *J. Chem. Phys.* **2024**, *160*, 054502.
[77] S. Wang, G. Liu, W. Wan, X. Li, J. Li, C. Wang, *Adv. Mater.* **2023**, *36*, 2306546.
[78] W. Deng, Z. Deng, Y. Chen, R. Feng, X. Wang, *Angew. Chem.* **2024**, *63*, e202316499.
[79] Y. Song, Y. Liu, S. Luo, Y. Yang, F. Chen, M. Wang, L. Guo, S. Chen, Z. Wei, *J. Mater. Chem. A* **2024**, *12*, 6572.
[80] A. Lahiri, P. Hirani, S. Haghani, F. Endres, *J. Electrochem. Soc.* **2024**, *171*, 012505.
[81] G. Wang, H. Fu, J. Lu, S. Huang, C. Pei, D. Min, Q. Zhang, H. S. Park, *Adv. Energy Mater.* **2024**, *14*, 2303549.
[82] M. Li, X. Zhu, C. Jiang, X. Liu, Z. Li, G. Xu, H. Wang, M. Wu, C. Song, W. Zhou, C. Wu, G. Wang, *Small* **2024**, *20*, 2402925.
[83] M. S. Kumar, H.-H. Shen, S. K. Batabyal, J.-Y. Lin, T.-W. Lin, *J. Energy Storage* **2024**, *88*, 111606.

[84] J. Li, S. Qin, M. Xu, W. Wang, J. Zou, Y. Zhang, H. Dou, Z. Chen, *Adv. Funct. Mater.* **2024**, *34*, 2402186.
[85] F. Wan, L. Zhang, X. Wang, S. Bi, Z. Niu, J. Chen, *Adv. Funct. Mater.* **2018**, *28*, 1804975.
[86] Q. Peng, R. Cao, H. Ma, Y. Li, E. Zhu, X. Zhu, N. Ma, M. Xue, X. Zhang, *J. Mater. Chem. A* **2025**, *13*, 6586.
[87] W. Yang, X. Du, J. Zhao, Z. Chen, J. Li, J. Xie, Y. Zhang, Z. Cui, Q. Kong, Z. Zhao, C. Wang, Q. Zhang, G. Cui, *Joule* **2020**, *4*, 1557.
